# Optimization of the sealed yellowing parameters and suitability evaluation of the different cultivars for manufacturing the Pingyang Huangtang tea

**DOI:** 10.1016/j.fochx.2025.102615

**Published:** 2025-06-03

**Authors:** Shanshan Wu, Dingwu Zhang, Siyi Hu, Cunyu Li, Zhanbo Dong, Yurong Hu, Fangyuan Fan, Jianhui Ye, Xinqiang Zheng, Yuerong Liang, Liaoyuan Yu, Jianliang Lu

**Affiliations:** a Zhejiang University Tea Research Institute, Hangzhou 310058, China; b KangShi (Shanghai) Food Science and Technology Co., Ltd., China; c Wanquan Town People's Government, Pingyang County, Wenzhou 325409, China; d Zhejiang Agricultural Technology Extension Center, Hangzhou 310020, China

**Keywords:** *Camellia sinensis*, Yellow tea, Aroma and taste attributes, Sealed yellowing, Cultivar, Manufacturing suitability

## Abstract

Lack of optimal sealed yellowing (SY) technique and suitable tea cultivars limits the yield and quality improvement of the Pingyang Huangtang (PYHT) tea. In this study, the effect of SY temperature, relative humidity (RH) and ventilation operation on the PYHT quality had been investigated. Results showed total sensory score was significantly influenced by SY temperature and RH, and optimal PYHT quality could be achieved when SY was performed at 60 °C, 70 % RH for 10 h with 2 ventilation operation. Quality formation of PYHT was mainly associated with degradation of chlorophylls, fluctuation of volatile with different odor notes, and decrease in level of galloylated catechins and the ratio of total polyphenols to amino acids. The quality of tea made from the shoots of ‘Zhenong 801’, ‘Zhongcha 108’, ‘Jiukengzao’ and ‘5–21’ was higher than that of the commonly used cultivar ‘Pingyang Tezao’, therefore, these cultivars were recommended to apply in PYHT industry.

## Highlights

• Temperature and RH of yellowing will significantly influence the PYHT quality.
• Yellowing should be performed at 60 °C, 70 % RH for 10 h with ventilation 2 times.
• Cultivar ZN801, ZC108, 5-21 and JKZ are much suitable to manufacture the PYHT.
• Technique defines the lower limit of PYHT quality and cultivar does the upper one.

## Graphical abstract

**Figure:**
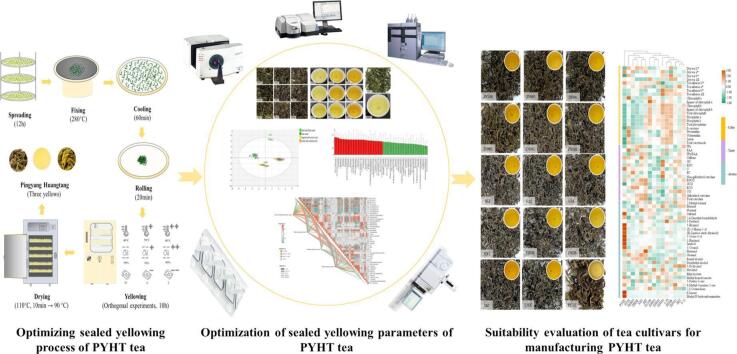

## 1 Introduction

Tea is one of the most consumed non-alcoholic beverages worldwide, it can be divided into six major types according to their manufacturing technologies, including green tea, oolong tea, black tea, dark tea, white tea and yellow tea (YT). YT is mainly produced in Hunan, Anhui, Zhejiang, Sichuan, Hubei and Guangdong Provinces of China (Li et al., 2024). YT has gained increasing attention for its sweet aroma and mellow taste as well as potential health benefits, such as anti-oxidation, anti-inflammation, anti-obesity, anti-cancer, anti-metabolic syndrome and gut microbiota regulation (Feng et al., 2024). YT has been considered as a type of lightly fermented tea, with unique “Three Yellows” characteristics *i.e.*, yellow dry tea, yellow tea infusion, and yellow infused leaves (Li, et al., 2024). Quality of tea sensory can comprehensively evaluated through examining the appearance of dry tea, infusion color, aroma, taste and infused leaves by discrimination of vision, olfaction, taste and touch. The characteristic flavor of YT is strongly correlated with the taste-related and the aroma-related chemical composition of tea leaves (Wei et al., 2021).

The quality of YT is mainly influenced by geography, cultivar, harvest season, plucking standard, and the manufacturing technology (Li, et al., 2024; Qin et al., 2024; Sheng et al., 2024; Wei et al., 2024). The basic manufacturing steps of YT includes the spreading, fixing, rolling, sealed yellowing and drying (Wang et al., 2019). Among them, sealed yellowing is the key and characteristic step to form the distinctive quality of YT. Comparatively, YT exhibits a sweeter and mellower flavors owing to the changes of sensory traits and chemical compositions under the thermochemical reactions of the tea dhool during sealed yellowing (Wei et al., 2021). Sealed yellowing is usually performed in a chamber or cloth bag, and many factors including moisture content of the dhool, temperature, duration, as well as relative humidity (RH) and ventilation conditions in the chamber, will affect the efficiency (Fan et al., 2022).

With an extension of the sealed yellowing time, mellow taste and sweet aroma of YT significantly increase to the peak values, then gradually decrease because of over-oxidation of the aroma and over-transformation of the taste, thus, it is very important for producing YT to seek a suitable sealed yellowing time (Fan et al., 2022; Wei et al., 2021). Wet- and dry- sealed yellowing can be usually adopted in the actual practice according to the moisture content of tea dhool. Compared with dry-sealed yellowing, wet-sealed yellowing will lead to more drastic physicochemical alterations in the tea dhool (Wei et al., 2020). The temperature of sealed yellowing profoundly influences the thermal reaction rate and yellowing progress of the dhool, higher temperature treatment will help to form the typical characteristics of yellow tea, while lower temperature treatment is beneficial for the formation of flavors similar to green tea rather than yellow tea (Wei et al., 2023). The measure of ventilation through turning over and dispersing during the yellowing process is also required to avoid the excessive accumulation of the heat and formation of the stuffy flavor, and the yellowing rate of tea dhool can be accelerated by decreasing the frequency of ventilation (Fan et al., 2022).

As the origin of tea plant in the world, China has abundant germplasms with different chemical compositions and distinctive tea-processing suitability. Chemical composition of various tea cultivars has been proven to be the prerequisite of suitability for manufacturing different types of tea, and thus different tea cultivars usually possess distinctive manufacturing suitability (Chen et al., 2022; Li et al., 2020). Tea cultivars with different leaf sizes and colors exhibit considerable variations in terpene index of the volatile compounds as well as in ratio of non-volatile compounds such as tea polyphenols / free amino acids (TPs/FAA) (Chen et al., 2022). Multiple studies have shown that cultivars also have a significant impact on formation of the aroma in other teas (Li et al., 2022; Wang et al., 2017). Moreover, the TPs/FAA has been considered as an index of taste balance, big TPs/FFA usually indicates the astringent and bitter taste while small one implies the umami and mellow tastes, thus, the TPs/FAA has also been used as a discriminant index tea-processing suitability for the cultivars (Chen et al., 2022).

Pingyang Huangtang (PYHT), regarded as a representative yellow tea of Zhejiang Province, China, is traditionally manufactured according to the wet-sealed yellowing technique (Feng, et al., 2024). To date, PYHT is usually produced using the shoots with one bud and two leaves mainly harvested from a local cultivar ‘Pingyang Tezao’. Unstable quality and low output are the key obstacles limiting the development of PYHT tea industry. The lack of evaluation criteria is an important issue in the difficulty of forming the sealed yellowing standard, which will finally lead to unstable quality of the product. Meanwhile, the lack of suitable cultivars results in limited harvesting time of fresh leaves, ultimately leading to short production time and low yield of PYHT. The aim of the present study is to comprehensively optimize the sealed yellowing process according to orthogonal experiments, to clearly reveal the mechanism of characteristic flavor formation, and to appropriately screen out suitable cultivars for manufacturing PYHT with high quality.

## 2 Materials and methods

### 2.1 Chemicals and standards

Epigallocatechin gallate (EGCG), gallocatechin gallate (GCG), (+) catechin (C), epicatechin (EC), gallocatechin (GC), epigallocatechin (EGC), catechin gallate (CG), epicatechin gallate (ECG), caffeine, chlorophyll *a* (Chl a), chlorophyll *b* (Chl b), neoxanthin (Neo), violaxanthin (Vio), lutein (Lut), *β*-carotene (Car) and cross-linked polyvinylpolypyrrolidone (PVPP), were all purchased from Sigma-Aldrich Corporation (St. Louis, USA). Pheophytin a (Py a), pheophytin b (Py b) were obtained from Shanghai Zhenzhun Biotechnology Co., Ltd. High Performance Liquid Chromatographic (HPLC) grade acetonitrile and methanol were ordered from Merck Inc. (Shanghai, China). Formic acid and acetic acid (HPLC grade) and deuterated guaiacol (≥ 99 % purity) were obtained from Aladdin Inc. (Shanghai, China). Ethanol, disodium hydrogen phosphate, potassium dihydrogen phosphate, folin-ciocalteu reagent, ninhydrin hydrate, stannous chloride, sodium carbonate and glutamic acid were of analytical grade and ordered from Sinopharm Chemical Reagent Co., Ltd. (Shanghai, China). SPME fiber manual sampling holder and divinylbenzene / carboxen / polydimethylsiloxane (DVB / CAR / PDMS, 50 / 30 μm, 1 cm) microextraction fiber were purchased from Supelco (Bellefonte, PA, USA). The purified water obtained from Wahaha Group Corporation (Hangzhou, China) was used for sensory evaluation, and highly pure water prepared by Barnstead™ GenPure™ water system (Thermo Fisher Scientific, USA) was used for analysis of tea ingredients.

### 2.2 Plant cultivation and sample preparation

Tea cultivars (6-year-old) were cultivated in the experimental garden of Zhejiang University Tea Research Institution located in Hangzhou (30°23′ N, 119°52′ E), Zhejiang, China, and maintained through the common agricultural practices. The newly sprouted shoots with one bud and two leaves were plucked from cultivar ‘Zhongcha 108’ in October 2022, and used in the tests for optimization of the sealed yellowing. The similar tender shoots were harvested in April 2023 from 15 cultivars including ‘Pingyang Tezao’, ‘Zhongcha 108’, ‘Zhenong 805’, ‘Zhenong 801’, ‘Zhenong 901’, ‘Zhenong 301’, ‘Zhenong 302’, ‘Jiukengzao’, ‘5–21’, ‘1–35’, ‘Xiaoxiang 1’, ‘Fuding Dabaicha’, ‘Zhenghe Dabaicha’, ‘Mei Zhan’ and ‘Fujian Shuixian’, and used in the tests for suitable cultivar screening.

PYHT was manufactured according to common processing technology, including spreading, fixing, cooling, rolling, sealed yellowing and drying. The 12 h-spread shoots were fixed in a hot pan at 280 ± 10 °C till the moisture content of the fixed shoots was around 45 %, which was similar to actual production of PYHT. The fixed leaves were cooled at room temperature for 1 h and then rolled till the rolled twig rate was around 80 %. The rolled leaves were then performed the sealed yellowing under different conditions, the total duration was set as 10 h which was proven to be a proper yellowing duration to form fruity and floral aromas instead of dull odor (Dong et al., 2023). Finally, the yellowed leaves were dried at 110 °C for 10 min, and then at 90 °C till the moisture content was below 6.5 %. The whole tea was used for sensory evaluation, and the tea sample was ground into powder (passing through 80 mesh) in a grinding mill (Shanghai Jingxin Industrial Development Co., Ltd., China) and used for physicochemical analysis.

Tests for optimization of the sealed yellowing: An L_9_(3^3^) orthogonal experimental design was adopted to obtain the optimal parameters of sealed yellowing including temperature (40 °C, 50 °C, 60 °C), RH in chamber (50 %, 70 %, 90 %) and ventilation frequency in 10 h (10, 6, 2). The levels of the three factors were selected according to the local tea factory in Pingyang country, as well as the previous research by our team members (Fan et al., 2022) and the literature (Wei et al., 2020). The tests were conducted according to an orthogonal table (Table S1) in triplicate. A green tea was manufactured according to the corresponding technology in which the rolled leaves were directly dried at 110 °C for 10 min and at 90 °C till the moisture content was below 6.5 %, and used as a control.

Tests for suitable cultivars screening: The harvested fresh leaves of different cultivars were spread, fixed, cooled and rolled, then yellowed under optimized condition, and dried. Similarly, green teas were also prepared using the corresponding technology, and used as control. Tests were carried out in triplicate for each cultivar.

Detailed information of PYHT tea samples was presented in Table S2.

### 2.3 Sensory evaluation

A panel of five expert panelists (Grade II or above) who were qualified for tea sensory evaluation was recruited according to Tea Sensory Evaluator (GZB 6-02-06-11). Tea samples were evaluated according to Methodology for Sensory Evaluation of Tea (GB/T 23776-2018) and Tea Vocabulary for Sensory Evaluation (GB/T 14487-2017). Briefly, the dry tea appearance was evaluated and scored according the color and shape in sample tray. After that, 3 g of yellow tea samples were brewed with 150 mL of boiling water in a tea cup with the lid on for 5 min (green tea control, for 4 min), and then the infusion was filtered to a matching tea bowl. The infusion color, aroma, taste, and infused leaves of the samples were sequentially evaluated, described and scored. A 100-point scoring system was used for evaluation according to the weight of factors including the appearance (25 %), infusion color (10 %), aroma (25 %), taste (30 %) and the infused leaves (10 %) of the yellow tea samples. Ethical permission was not required, and the panelists gave their consent to take part in the sensory evaluation and use their information.

### 2.4 Measurement of color difference

A Color Quest XE colorimeter (Hunter Lab, Virginia, USA) was employed to evaluate the color difference indices of the dry tea and infusion. These indices included the L*, a*, b* and ΔE values which represented lightness, red-green degree, yellow-blue degree, and total color difference, respectively. Detailed performance was carried out according to the previous paper (Li et al., 2024).

### 2.5 Determination of non-volatile compounds

Moisture content of the tea sample was measured according to method described in GB/T 8304-2013. Briefly, 5.000 g tea samples were dried at 103 ± 2 °C until constant weight (m) had been achieved, and moisture content could be calculated as (5.000-m)/5.000*100 %. Total polyphenols (TPs) of tea sample were quantified using folin-ciocalteu colorimetric method as described in GB/T 8313-2018, free amino acids (FAA) of tea sample were quantified using ninhydrin colorimetric method as described in GB/T 8314-2013.

Photosynthetic pigments and their derivatives of the tea sample, including Chl a, Chl b, chlorophyll *a*’ (Chl a’), chlorophyll *b*’ (Chl b’), Py a, Py b, Neo, Vio, Lut and Car, were extracted with pre-cooled acetone/water (9/1, v/v) and monitored according to a HPLC method as previously reported (Li et al., 2019), the contents of Chl a’ and Chl b’ were quantified using Chl a and Chl b as standards, respectively. Catechins and caffeine were extracted with 50 % aqueous ethanol from the tea sample and detected through a HPLC method described as previous paper (Li et al., 2019). The target compound was qualified and quantified with the retention time and peak area of the external standard.

### 2.6 Detection of volatiles

Volatile compounds were extracted from tea samples through headspace- solid phase microextraction (HS-SPME). Ground tea sample (1.00 g) was mixed with 50 mL highly purified water (70 °C), and extracted at 70 °C for 10 min in a water bath (Shenshun, Shanghai, China). The tea infusion was centrifuged at 25 °C and 5000 rpm for 10 min after being cooled to room temperature, 10 mL supernatant was transferred into a 50-mL headspace vial. After 100 μL of internal standard solution (20 μg/mL deuterated guaiacol) and 2.0 g of sodium chloride (fully dried) were added, the vial was immediately sealed and incubated at 40 °C in a water bath, then the SPME fiber was inserted at 0.5 cm above the liquid surface and extracted for 1.0 h.

The volatile compounds on SPME fiber were detected in the splitless mode on a QP2010 Shimadzu gas chromatography – mass spectrometry (GC–MS) (Kyoto, Japan) equipped with HP-INNOWax capillary column (30 m × 0.25 mm, 0.25 μm, Agilent, USA). The high-purity helium (99.999 %) was used as carrier gas, at a flow rate of 1.0 mL/min. The injection port temperature was maintained at 200 °C injection port, and the fiber was inserted injection port and desorbed for 5 min. The time program of column temperature was set as follows: initially holding at 50 °C for 10 min, increasing to 150 °C at ramp rate of 3 °C/min and holding at 150 °C for 1 min, then increasing to 230 °C at ramp rate of 15 °C/min and holding at 230 °C for 3 min. After separation, the volatiles were ionized through electron ionization (EI) mode with an energy of 70 eV, and the MS scanning range was 35–400 *m*/*z*. Substance identification was carried out according to comparison of the obtained mass spectrometry data with the those of standard substances provided by NIST (2020) spectrum library using a criterion of similar retention index and match factor larger than 85 %. Detailed performance was carried out according to the previous paper (Hu et al., 2018). Relative content of the identified compounds was obtained according to Eq. (1).

(1) $$\text{Relative content of target}\;\left(\mathrm{\mu g}/g\right)=\frac{\left(\text{Peak area of the target}/\text{peak area of internal standard}\right)\times 2\mathrm{\mu g}}{\text{Amount of}\;\mathrm{tea}\;\left(g\right)\times \left(1-\mathrm{MC}\right)\times \left(10\mathrm{mL}/50\mathrm{mL}\right)}$$

where the MC indicated the moisture content of the tea sample.

The ratio of the target volatile relative content (C_i_) to its odor threshold (OT) was calculated and used as the relative odor activity values (ROAVs) of the target, in order to assess the contribution of each volatile to the overall aroma profile in the samples. In general, a volatile was typically considered as potential contributor of aroma when its ROAV was larger than 1.

### 2.7 Statistical analysis and figure preparation

All the data were present as mean ± SD (Standard deviation) of the three repeats. One-Way ANOVA and Duncan's post-hoc test were carried out on the SPSS Statistics 27.0 (IBM, Chicago, IL, USA), and *p* < 0.05 was considered as significant difference. Orthogonal partial least squares-discriminant analysis (OPLS-DA) was performed by using SIMCA 14.1 software (Umetrics, Umea, Sweden). The heatmap, mantel test correlation, histogram and correlation visualizations were constructed using TBtools (Chen et al., 2023), Chiplot.online, Origin 2024b (Origin Lab Inc., Northampton, MA, USA), respectively.

## 3 Results and discussion

### 3.1 Optimization of sealed yellowing parameters

#### 3.1.1 Effect of sealed yellowing on sensory of PYHT tea

**Fig. 1:**
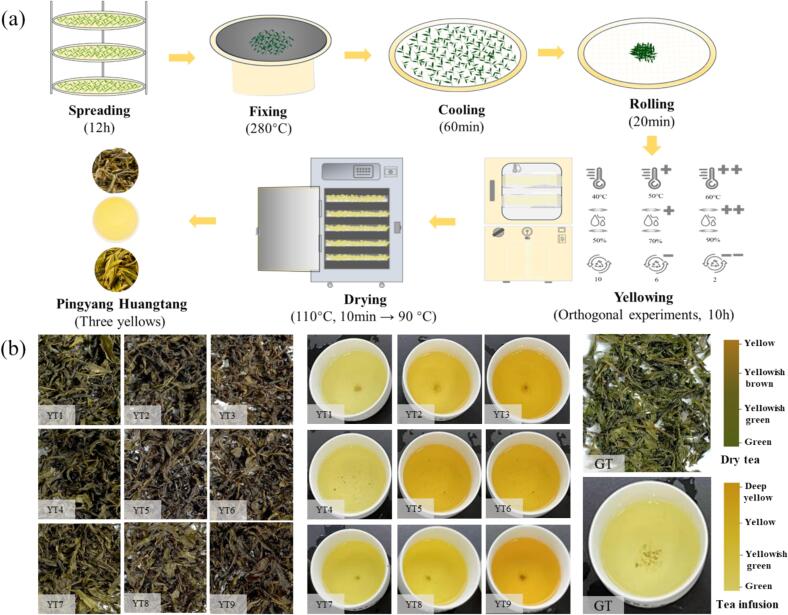
Optimizing the sealed yellowing process of PYHT tea. (a) Procedure for manufacturing the PYHT tea; (b) Dry tea appearance and infusion color of PYHT tea (YT1-YT9) manufactured under different sealed yellowing conditions. GT, green tea (control). (For interpretation of the references to color in this figure legend, the reader is referred to the web version of this article.)

According to the orthogonal experimental design with 3 factors and 3 levels, 9 treatments (27 samples) were carried out under different sealed yellowing conditions for 10 h (Fig. 1a). Sensory evaluation was performed by panel and the results had been shown in Fig. 1b and Table 1. In comparison with the green tea without the sealed yellowing step, all the PYHT samples exhibited somewhat characteristics of yellow tea, in particular, color of the dry tea, tea infusion and infused leaves turned yellow, some “sweet” attributes appeared in the aroma and taste. Among the 9 treatments, total scores of the YT3, YT6 and YT9 were relatively close and higher than 90, although the YT2 and YT7 exhibited higher scores of dry tea appearance. Appropriate increase in the ventilation frequency and the RH could significantly improve the quality of yellow tea, which was consistent with previous report (Fan et al., 2022). In the YT3, YT6, and YT9 treatments, their temperature conditions were same (60 °C), although the levels of RH and ventilation frequency were different. This implied that the temperature was the most important among the 3 factors of the sealed yellowing, specially, sealed yellowing at high temperature (60 °C) would be beneficial for the formation of yellow tea quality. In order to intuitively obtain the optimal parameters of the sealed yellowing, the range and contribution rate of the different factors and levels were calculated. Except the appearance score was greatly affected by ventilation frequency, the scores of all other quality indicators were remarkably influenced by the temperature, followed by RH. The effect of RH was also found in previous researches in which sweet and floral aromas as well as the yellowness of infusion and sweetness of taste were found significantly increased after RH was adjusted from 40–60 % to 67 % during optimization of the yellowing process (Wei et al., 2024; Wei et al., 2023). The optimal combinations for the appearance, infusion color, aroma, taste, infused leaves and total quality were A2B1C3, A3B2C1/3, A3B2C1, A3B3C3, A3B1/2/3C1/2/3 and A3B2C3, respectively (Table S3), suggesting that effect of these 3 sealed yellowing factors varied with the different quality indicators, in particular, besides high temperature was beneficial for most quality indicators, medium RH was in favor of the aroma, while high one would benefit to taste. For total score, the temperature (*p* = 0.000) was the most important factor, followed by the RH (*p* = 0.014), but ventilation frequency (*p* = 0.975) was not a significant factor (Table S4). Appropriately increasing ventilation could speed up the degree of yellowing, and together with RH, affected the quality of yellow tea (Fan et al., 2022). Although insignificant effect of the ventilation frequency, considering the operation convenience of less frequency of ventilation, the A3B2C3 was recommended as the optimal combination for total quality of the PYHT tea, in which the sealed yellowing was performed at 60 °C and 70 % RH for 10 h with 2 ventilation operation. Optimal sealed yellowing parameters differed probably depending on factors such as yellowing duration, yellowing frequency and tenderness of fresh tea leaves.

**Table 1:** Sensory results of the PYHT teas prepared with different sealed yellowing treatments.

Treatments	Appearance (25 %)		Infusion color (10 %)		Aroma (25 %)		Taste (30 %)		Infused leaves (10 %)		Total score
	Description	Score	Description	Score	Description	Score	Description	Score	Description	Score	
YT1	yellowish green	89	yellowish green, brilliant yellow	86	faint scent	88	umami and fresh	85	yellowish green	85	87.10
YT2	yellow and bright, slightly glossy	91	yellow, bright	87	slightly sweet and fragrant	89	mellow	88	greenish yellow	87	88.57
YT3	yellowish brown	90	deep yellow, brilliant bright	90	sweet and fragrant, slightly stuffy	91	mellow, slightly sweet	91	brownish yellow	89	90.45
YT4	yellowish green	89	yellowish green, bright	88	faint scent with slightly sweet	89	mellow, slightly astringent	86	yellowish green	85	87.85
YT5	yellow，a few glossy	90	yellow, bright	91	slightly sweet and fragrant	89	mellow	88	greenish yellow	87	88.90
YT6	yellowish brown	90	orange, brilliant bright	93	sweet and cooked	92	mellow	90	brownish yellow	89	90.73
YT7	yellowish green，slightly glossy	91	yellowish green, bright	86	faint scent	86	slightly mellow, fresh	89	yellowish green	85	87.67
YT8	yellow	89	yellow, bright	88	slightly sweet and cooked	89	mellow, a few sweet	90	greenish yellow	87	89.12
YT9	yellowish brown	88	yellow, bright	90	sweet and slightly cooked	90	sweetish mellow	92	brownish yellow	89	90.50
Green tea	fresh green，slightly glossy	–	light green, brilliant bright	–	fresh and faint scent	–	umami and fresh, mellow	–	green, bright	–	–

Note: The treatment represented combination of different temperature (40 °C, 50 °C, 60 °C), RH (50 %, 70 %, 90 %) and ventilation frequency (10, 6, 2) during sealed yellowing process which was indicated in Table S1.

#### 3.1.2 Effect of sealed yellowing on color and pigments of PYHT tea

**Fig. 2:**
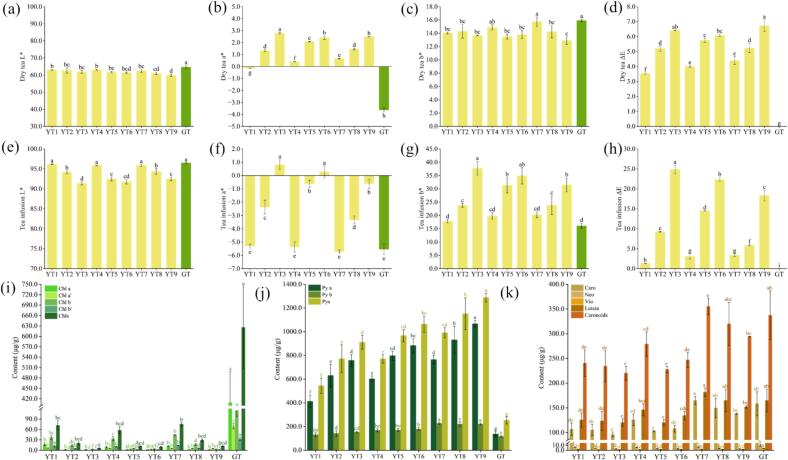
Color difference and contents of pigments in PYHT tea manufactured under different sealed yellowing conditions. (a) L* value of dry tea; (b) a* value of dry tea; (c) b* value of dry tea; (d) ΔE value of dry tea; (e) L* value of tea infusion; (f) a* value of tea infusion; (g) b* value of tea infusion; (h) ΔE value of tea infusion; (i) Contents of chlorophylls; (j) Contents of pheophytins; (k) Contents of carotenoids. Different letters indicated significant difference at *p* < 0.05.

The colors of dry tea, tea infusion, and infused leaves were essential for tea sensory quality, also regarded as indicators to distinguish different types of tea (Wei et al., 2023). The color of dry tea and infusion differed as the sealed yellowing parameters changed (Fig. 1b). In comparison with the green tea, significantly decreased L* and b* values as well as increased a* and ΔE values were observed in the dry PYHT teas prepared under different sealed yellowing conditions, similar but different, significantly decreased L* but increased a*, b* and ΔE values were witnessed in the infusions of PYHT teas (Fig. 2a-2h). Along with an increase in temperature of sealed yellowing, dry tea and infusion exhibited a slight decrease in L* value but a dramatic increase in a* and ΔE values, as well as a reverse change in b* value. Along with increase in RH, decreased L* value as well as increased a*, b* and ΔE values were observed in dry tea, but the least L* value as well as the highest a*, b* and ΔE values were witnessed in tea infusion. The a*, b* and ΔE values similarly increased with a decrease in ventilation frequency. In addition, the a* value was dramatically influenced by the sealed yellowing, especially the temperature (Table S5). It was clear that the sealed yellowing would lead to a decreased brightness and greenness and to a deepened color of dry tea and infusion, indicating the noticeable yellowing of the PYHT appearance might be mainly due to reduction of compositions with green color instead of increment of ingredients with yellow color.

Chlorophylls and carotenoids were the main lipid-soluble pigments in tea leaves and significantly impacted on the color of the raw materials and end products (Chen, Han, He, Cheng, & Tong, 2025;Li, Jin, Sun, Ye, & Liu, 2019). The content and proportion of pigments in tea leaves varied with cultivars and processing techniques (Li, et al., 2022; Wen et al., 2025). To further explore the color formation of PYHT after different yellowing treatments, chlorophylls and the derivatives as well as the carotenoids were quantified through HPLC. In comparison with the green tea, significantly lower contents of Chl a, Chl a’, Chl b, Chl b’ and total chlorophylls (Chls), but higher contents of Py a, Py b and total pheophytins (Pys), while relatively slight variations of the carotenoids were witnessed in PYHT teas (Fig. 2i-2j). In all PYHT samples, *β*-carotene and lutein were the major components of the carotenoids, while the neoxanthin and violaxanthin were the minor ones (Fig. 2k), which was consistent with previous report (Li, Jin, Sun, Ye, & Liu, 2019). Thus, *β*-carotene and lutein might also play an indispensable role in contributing to the color of dry tea and infused leaves, besides the role as an important precursor of volatiles. Along with an increase in the temperature of sealed yellowing, the contents of chlorophyll and carotenoid components decreased significantly, while the contents of pheophytin components increased remarkably in PYHT teas. The contents of pheophytins and carotenoids increased with the RH. Meanwhile, the pigments, especially carotenoids, varied less along with variation of ventilation frequency (Table S6). It was clear that conversion from chlorophylls to pheophytins dramatically took place during the sealed yellowing and significantly fluctuated with the temperature and humidity, while the carotenoids also changed but in a relatively small range. This implied that the chlorophylls rather than the carotenoids were susceptible to the muggy environment, especially at the high temperature, the color change during sealed yellowing was closely related to the degradation of the chlorophylls. Report showed that decreased chlorophylls and carotenoids as well as increased pheophytins were observed during manufacturing of the large-leaf yellow tea (Li et al., 2024), suggesting that chlorophylls conversion might commonly occur during manufacturing of yellow tea, especially in the sealed yellowing step, regardless of the distinctive parameters of the step were adopted.

#### 3.1.3 Effect of sealed yellowing on taste-related components of PYHT tea

**Fig. 3:**
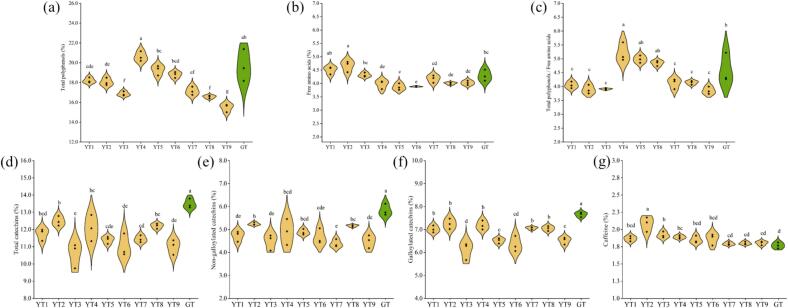
Taste-related components in PYHT tea manufactured under different sealed yellowing conditions. (a) Content of total tea polyphenols; (b) Content of free amino acids; (c) Value of TPs/FAA; (d) Content of total catechins; (e) Content of non-galloylated catechins; (f) Content of galloylated catechins; (g) Content of caffeine. Different letters indicated significant difference at *p* < 0.05.

The taste-related components in PYHT teas with different sealed yellowing treatments were analyzed and summarized in Fig. 3. Lower content of TPs, total catechins, galloylated catechins and non-galloylated catechins but high level of caffeine was witnessed in PYHT teas prepared under different sealed yellowing conditions in comparison with green tea, while the content of FAA varied with the sealed yellowing treatment. Along with an increase in temperature, the level of TPs, FAA, TPs/FAA and galloylated catechins decreased, the content of total catechins, non-galloylated catechins and caffeine followed a trend of first increasing and then decreasing (Table S7). Increased C, CG, GC and GCG were witnessed at higher temperature, which was in line with a report on Huoshan Huangya tea (Wei et al., 2023). The highest TPs and TPs/FAA were witnessed in the samples treated at 70 % RH, while the highest FAA was done in samples treated at 50 % RH, however, the catechins changed in a small range among the different treatment of RH. With decreasing the ventilation frequency, the level of TPs, FAA, total catechins, galloylated catechins, non-galloylated catechins and caffeine followed a trend of first increasing and then decreasing (Table S7). It should be mentioned that the change of catechins was largely dependent on the temperature, while the variation of the TPs/FAA and FAA was mainly subjected to the RH, indicating that catechins were sensitive to heat treatment and their content decreased at high temperatures probably through degradation or polymerization, while amino acids were susceptible to the RH and their level fluctuated at different RH probably through protein hydroxylation and carbonyl-amine reaction (Yin et al., 2023).

#### 3.1.4 Effect of the sealed yellowing on the volatiles of PYHT tea

**Fig. 4:**
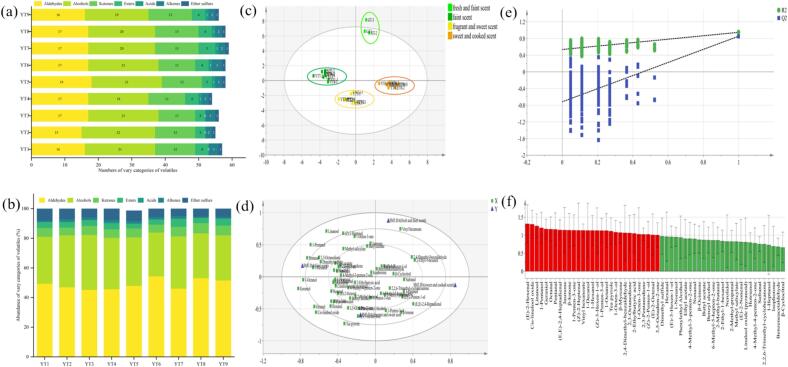
The profile of volatiles in PYHT tea prepared under different sealed yellowing conditions. (a) Numbers of volatiles in each category; (b) Abundance of volatiles in each category; (c) The score plots of OPLS-DA (R^2^Y = 0.956, Q^2^ = 0.859); (d) The loading scatter plot of OPLS-DA; (e) The cross-validation by a 200-times permutation test (R^2^ = 0.535, Q^2^ = -0.718); (f) The key volatiles (with VIP > 1) indicated by red column. (For interpretation of the references to color in this figure legend, the reader is referred to the web version of this article.)

A total of 68 volatiles were identified by GC/MS from the PYHT teas manufactured under different sealed yellowing conditions (Table S8), including 24 alcohols, 19 aldehydes, 15 ketones, 5 esters, 3 alkenes, 1 acid, and 1 other (Fig. 4a). Among them, aldehydes and alcohols were the major volatiles, accounting for more than 80 % of the total amount (Fig. 4b and Table S9). Compared with green tea, decreased (Z)-2-heptenal (with apple-like, vegetable-like note), 1-octen-3-one (with mushroom note) and (Z)-3-hexen-1-ol (with green, leafy, grassy notes), but increased (E)-2-hexenal (with green, leafy, fruity notes), (E)-2-octenal (with fatty, herbal, cucumber-like notes), (E, E)-2,4-heptadienal (with fatty, nutty, hay, green notes), tea pyrrole (with nutty and sweet notes), (E)-3-hexen-1-ol (with green, leafy, grassy notes), linalool oxide (furanoid) (with sweet, floral, creamy notes), phenylethyl alcohol (with floral, rose-like notes), *β*-ionone (with floral, woody, sweet, fruity, berry notes), 1-penten-3-ol (with fatty and mushroom notes), (Z)-2-penten-1-ol (with green and fruity notes), 1-penten-3-one (with pungent note), 6-methyl-5-hepten-2-one (with popcorn-like note), and 3,5-octadien-2-one (with creamy and fruity notes), were witnessed in all PYHT samples. It seemed that volatiles with floral and fruity aroma properties increased their contents but those with grassy properties decreased after yellowing treatments. However, the level of all the chemical categories of volatiles decreased along with an increase in temperature, meanwhile, relatively higher level of aldehydes and lower level of alcohols were retained in the PYHT teas processed under high RH and ventilation frequency conditions (Table S10), suggesting that many volatiles would be newly formed during sealed yellowing, and yellowing at high temperature might result in significant loss of volatiles but help to shape high quality, RH and ventilation frequency would influence the aldehydes and alcohols in a converse manner.

The OPLS-DA was employed to establish a linkage between characteristic volatiles and aroma type classification of PYHT samples and the control green tea. The established OPLS-DA model, with R^2^Y = 0.956 and Q^2^ = 0.859, exhibited a typical fitness and predictive capability. In the score plots of OPLS-DA, the PYHT teas and green tea (control) could be well distinguished into 4 types, including “fresh and faint scent”, “faint scent”, “fragrant and sweet scent” and “sweet and cooked scent” (Fig. 4c). The volatiles of these 4 aroma types were mainly distributed sequentially in the first, second, third and fourth quadrant. According to loading scatter plot of the model (Fig. 4d), the vinyl hexanoate, 2,4-dimethyl-benzaldehyde and 2-ethyl-1-hexanol were the representative aroma substances of “fresh and faint scent”, the linalool, 1-pentanol, pentanal and 1-hexanol were the representative aroma substances of “faint scent”, while the 1-octanol, geraniol, octanal, cis-linalool oxide and tea pyrrole were the main indicators of “fragrant and sweet scent”, similarly, the *β*-ionone and (E, E)-2,4-heptadienal were the main indicators of “sweet and cooked scent”. The cross-validation (*n* = 200, R^2^ = 0.535, Q^2^ = −0.718) presented the feasibility of the established OPLS-DA model (Fig. 4e). Twenty-nine key volatiles were totally screened out and considered as important variables to distinguish the aroma types of the different PYHT tea based on variable importance in the projection (VIP) >1 (Fig. 4f). Among these volatiles, eleven compounds including linalool, 1-pentanol, geraniol, pentanal, (E, E)-2,4-heptadienal, *β*-ionone, 1-penten-3-one, 1-octanol, 2,4-dimethyl-benzaldehyde, 1-Octen-3-one, and (E)-2-octenal were considered as the key differential substances contributing to the aroma quality because their ROAV values were larger than 1 (Table 2). Obviously, after sealed yellowing treatment, the green leaf odor gradually weakened, while the fruity, sweet and fatty aroma clearly enhanced, leading to form the aroma perception of yellow tea.

**Table 2:** Relative odor activity values of the key volatiles screened out from PYHT tea manufactured under different sealed yellowing conditions.

Volatiles	VIP	Odor threshold (μg/L)	Odor descriptions	Relative odor activity values								
				YT1	YT2	YT3	YT4	YT5	YT6	YT7	YT8	YT9
(E)-2-Hexenal	1.32	17^a^	Green, leafy, fruity	0.25	0.17	0.13	0.30	0.21	0.13	0.35	0.26	0.20
Cis-linalool oxide	1.30	50^b^	Flower	0.66	0.66	0.49	0.78	0.58	0.40	0.85	0.47	0.43
Linalool	1.26	0.22^a^	Floral, sweet, grape-like, woody	390.51	287.58	237.76	368.12	229.01	151.70	538.84	285.63	230.06
1-Pentanol	1.20	3.9^b^	Balsamic	7.35	4.58	3.49	5.88	3.45	2.70	5.10	2.96	2.37
Geraniol	1.17	7.5^a^	Rose-like, sweet, honey-like	12.98	12.96	12.89	13.97	11.41	9.83	14.22	10.97	9.84
Octanal	1.17	0.7^c^	Pungent, slightly fragment	4.66	5.08	4.28	3.87	3.89	2.85	2.86	0.00	0.00
Pentanal	1.16	8^b^	Almond, malt	14.42	7.78	6.63	11.49	9.18	6.17	11.59	7.91	4.63
(E, E)-2,4-Heptadienal	1.15	0.032^c^	Nust, fat, green	665.90	1334.60	944.62	1084.90	1098.84	1222.86	1649.06	2353.54	1644.00
Jasmone	1.14	7^a^	Floral, woody, herbal, citrus, jasmine-like	0.21	0.23	0.00	0.30	0.00	0.25	0.29	0.20	0.22
*β*-Ionone	1.14	0.01^d^	Violet-like, raspberry, floral	550.56	559.71	626.89	570.59	714.61	643.30	767.30	780.61	918.59
1-Penten-3-one	1.14	1^b^	Pungent	12.08	13.23	10.68	16.57	14.64	12.81	27.70	26.71	16.03
(Z)-2-Heptenal	1.14	n.f.	n.f.	–	–	–	–	–	–	–	–	–
Vinyl hexanoate	1.14	n.f.	n.f.	–	–	–	–	–	–	–	–	–
1-Decanol	1.13	775^d^	Fatty, waxy	0.00	0.00	0.00	0.00	0.00	0.00	0.00	0.00	0.00
1-Hexanol	1.13	5.6^a^	Green, grassy	0.81	0.92	0.81	0.82	0.66	0.44	0.80	0.47	0.46
(Z)-3-Hexen-1-ol	1.13	70^c^	Green, leafy, grassy	0.00	0.10	0.08	0.00	0.11	0.06	0.11	0.07	0.04
1-Penten-3-ol	1.13	400^d^	Green	0.01	0.01	0.01	0.01	0.01	0.01	0.01	0.02	0.02
1-Octanol	1.13	0.022^a^	Green, citrus, fatty, coconut-like	656.47	717.25	570.03	732.35	559.61	442.98	650.07	530.11	368.24
Tea pyrrole	1.11	100^e^	Burnt, roasted odor	0.02	0.03	0.04	0.03	0.03	0.02	0.02	0.02	0.03
1-Octen-3-ol	1.08	1^c^	Earthy, green, oily, vegetative-like, fungal	3.33	4.07	3.70	4.30	4.65	2.72	3.62	3.27	2.31
*β*-Myrcene	1.07	15^d^	Geranium-like, carrotlike	0.35	0.76	0.59	0.69	0.71	0.40	0.61	0.37	0.37
2,4-Dimethyl-benzaldehyde	1.06	0.2^f^	Bitter almond flavor	1441.43	1268.68	1077.07	1219.68	1112.53	1481.58	1472.95	1602.10	1506.76
2,3-Octanedione	1.06	3^g^	Milk, buttery, cooked tasting	3.40	1.69	1.26	2.19	1.72	1.13	2.13	1.77	0.83
2-Ethylbutyric acid	1.05	n.f.	n.f.	–	–	–	–	–	–	–	–	–
1-Octen-3-one	1.03	0.04^h^	Mushroom	41.13	42.34	24.71	53.20	38.61	26.70	44.65	43.27	23.24
2,3-Pentanedione	1.02	30^h^	Buttery, creamy	0.03	0.03	0.02	0.04	0.03	0.02	0.05	0.04	0.03
(Z)-2-Penten-1-ol	1.02	720^d^	Green, fruity	0.00	0.00	0.00	0.00	0.00	0.00	0.01	0.01	0.01
(E)-2-Octenal	1.01	3^a^	Sweet, green, citrus, fatty, herbal	2.01	1.86	2.34	2.75	2.13	2.13	3.21	2.65	1.45
3,5-Octadien-2-one	1.00	100^c^	Creamy, fruity	0.01	0.02	0.02	0.04	0.02	0.02	0.02	0.02	0.01

Note: The treatment was same as Table S1. The key volatiles were screened out according to VIP (variable importance in the projection) >1. Odor threshold data obtained from: a. Guo, Schwab, Ho, Song, & Wan, 2022; b. Zhou et al., 2023; c. Chen et al., 2024; d. Xie et al., 2023; e. Zhai et al., 2023; f. Xu et al., 2022; g. Wei et al., 2023; h. Majcher, Klensporf-Pawlik, Dziadas, & Jeleń, 2013; n.f., not found.

#### 3.1.5 Correlation assessment of sensory evaluation scores *versus* physicochemical indices

**Fig. 5:**
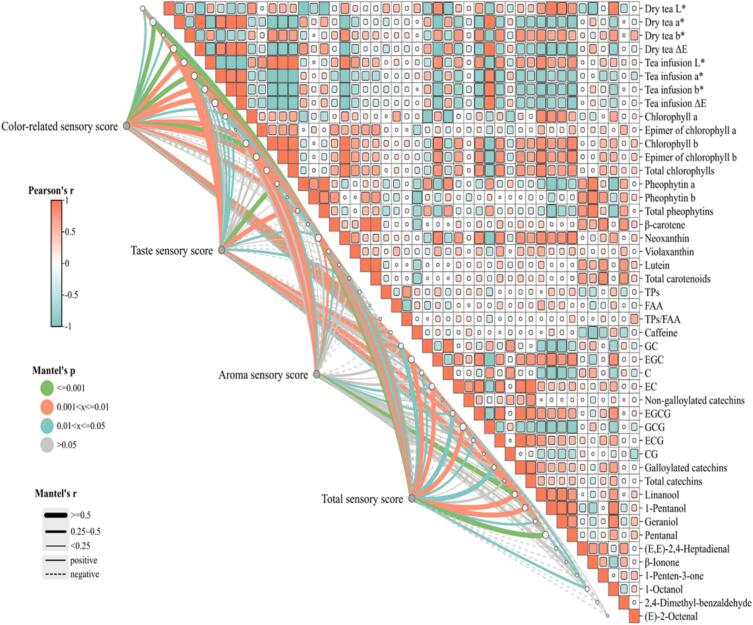
Correlation analysis between sensory scores and physicochemical indices including color, aroma, and taste. TPs, total polyphenols; FAA, free amino acids; C, (+) catechin; EC, epicatechin; GC, gallocatechin; EGC, epigallocatechin; CG, catechin gallate; ECG, epicatechin gallate; GCG, gallocatechin gallate; EGCG, epigallocatechin gallate.

The content of non-volatile and key volatile compounds was calculated and presented in Fig. 5. Significantly positive contribution of Chl a and Neo, and negative contribution of Py a, Py b and Pys were found to the L* value of dry tea. The level of Chl b, Chl b’, Chls, and Neo was positively correlated with the b* value of dry tea and L* value of tea infusion, while negatively correlated with the a* value of dry tea. Py a level was correlated positively with the a* value of dry tea, and a positive contribution of Chl a’ and Vio to the infusion L* value was also observed (*p*<0.05). In other words, the color difference, as an intuitive indicator of tea, was closely related to the color contributing substances. The color related sensory score (dry tea, tea infusion and infused leaves) was influenced significantly and positively by the L*, a*, b* and ΔE values of the dry tea and tea infusion, as well as the levels of Chl a, Chl b, Chl b’, Chls, and Neo. Significantly positive correlation was found between the taste sensory score and the content of EGC, GCG, and TPs. Astringency and bitterness of the infusion were mainly attributed to TPs, among which, gallaoylated catechins (EGCG, ECG, GCG, and CG) might be the key active components (Zhang, Cao, Granato, Xu, & Ho, 2020). The aroma sensory score was significantly and positively impacted by linalool which was considered as an aroma noted with sweet and floral of yellow tea (Wei et al., 2024). The total sensory score was correlated positively with most of the color difference indices of the dry tea and tea infusion, as well as level of pigments including Chl a, Chl b, Chl b’, Chls, Py a, Pys, Neo and Vio; meanwhile, gallaoylated catechins and catechin monomers except CG, along with the key volatiles such as pentanal, linalool, 1-pentanol, geraniol and 1-octanol positively contributed to the total sensory score (*p*<0.05).

### 3.2 Cultivars suitable for manufacturing the PYHT tea

#### 3.2.1 Sensory quality of PYHT tea processed from different cultivars

**Fig. 6:**
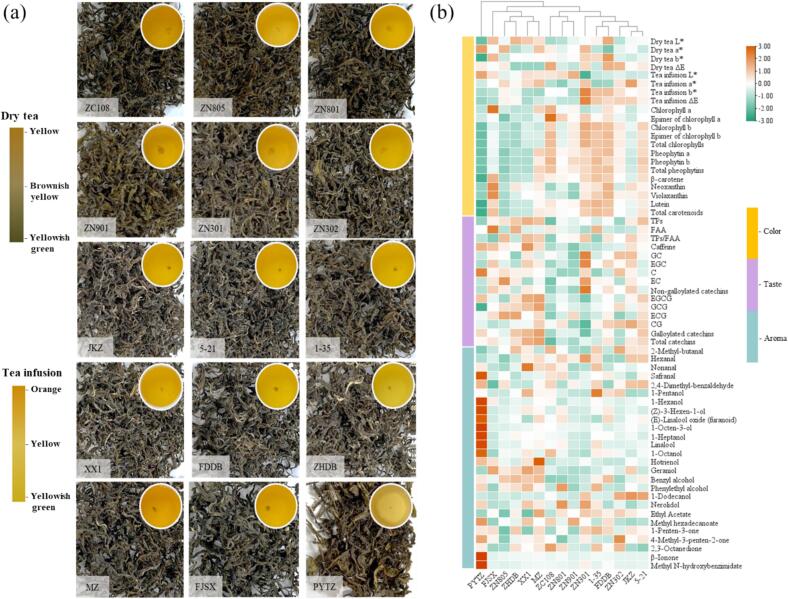
Dry tea appearance, infusion color and chemical profiles of PYHT tea manufactured from the shoots of different cultivars. (a) Dry tea appearance and infusion color of PYHT tea; (b) A heatmap of identified differential color, taste- and aroma-related chemical indices. TPs, total polyphenols; FAA, free amino acids; C, (+) catechin; EC, epicatechin; GC, gallocatechin; EGC, epigallocatechin; CG, catechin gallate; ECG, epicatechin gallate; GCG, gallocatechin gallate; EGCG, epigallocatechin gallate.

Different tea cultivars usually differ in the manufacturing suitability (Chen et al., 2022). Fresh shoots were harvested from 15 different cultivars and used to prepare the PYHT tea using the optimized sealed yellowing parameters. Although these teas exhibited typical quality traits of yellow tea, with “yellow appearance, infusion and infused leaves” and “sweet and mellow flavors”, obvious differences existed in the appearance, infusion color, aroma, taste and infused leaves among them (Fig. 6a), implying processing techniques can greatly shape the tea quality, but cultivars can also have a significantly influence. The tea would be considered as high quality and given high score when it possessed yellow and slightly glossy appearance, yellow and brilliantly bright infusion, heavily sweet and cooked aroma, umami and sweetish mellow taste, yellow and bright infused leaves (Table 3). Traditionally, the PYTZ was considered as a cultivar suitable for manufacturing the PYHT tea, when using the total score not less than the PYTZ as an admission standard, 10 cultivars were suitable for producing the PYHT tea, among them, the ZN801, ZC108, 5–21 and JKZ would be preferentially recommended since the total score of teas made from these cultivars was higher than that from others.

**Table 3:** Sensory evaluation of PYHT tea prepared from the shoots of different cultivars.

Cultivars (Clones)	Appearance (25 %)		Infusion color (10 %)		Aroma (25 %)		Taste (30 %)		Infused leaves (10 %)		Total score
	Description	Score	Description	Score	Description	Score	Description	Score	Description	Score	
ZC108	yellowish green	87	yellow, brilliantly bright	92	heavily sweet and cooked	92	sweetish mellow	92	yellow, bright	91	90.65
ZN805	brownish yellow	86	yellow, bright	91	sweet and cooked	91	sweetish mellow	91	yellow, brilliantly bright	92	89.85
ZN801	yellow, slightly glossy	91	yellow, slightly bright	90	sweet and cooked	91	umami, sweetish mellow	93	yellow, bright	91	91.50
ZN901	brownish yellow	87	yellow, brilliantly bright	92	sweet, slightly woody	89	sweetish mellow, slightly woody	89	yellow, bright	91	89.00
ZN301	brownish yellow	87	orange, slightly bright	89	slightly stuffy	89	stuffy	88	yellow, slightly bright	90	88.30
ZN302	brownish yellow	87	orange, brilliant bright	91	sweet and cooked	91	sweetish mellow	91	yellow, slightly bright	90	89.90
JKZ	yellowish green，slightly glossy	88	yellow, brilliantly bright	92	sweet and cooked	91	mellow, rich	90	yellow, bright	91	90.05
5–21	yellowish green，glossy	90	yellow, bright	91	heavily sweet and cooked	92	umami, slightly mellow	89	yellow, weakly bright	88	90.10
1–35	brownish yellow	86	yellow, bright	90	pure and normal	89	strong, astringent	88	yellow, weakly bright	88	87.95
XX1	yellowish green	87	yellow, slightly bright	89	sweet and cooked	91	sweetish mellow	91	yellow, slightly bright	90	89.70
FDDB	yellowish green	87	yellowish green, slightly bright	89	slightly ripe and stuffy	88	slightly bitter and astringent, stuffy	87	yellow, weakly bright	88	87.55
ZHDB	yellowish green	87	yellow, bright	90	heavily sweet and cooked	92	mellow	90	yellow, bright	91	89.85
MZ	yellowish green	87	yellow, bright	90	sweet and cooked	91	sweetish mellow	91	yellow, weakly bright	88	89.60
FJSX	yellowish green	87	yellow, bright	90	pure and normal	89	mellow	90	yellow, weakly bright	87	88.70
PYTZ	yellowish green	87	yellow, brilliantly bright	91	pure and normal	90	mellow	90	slightly yellow, weakly bright	85	88.85

Note: ZC108, ‘Zhongcha 108’; ZN805, ‘Zhenong 805’; ZN801, ‘Zhenong 801’; ZN901, ‘Zhenong 901’; ZN301, ‘Zhenong 301’; ZN302, ‘Zhenong 302’; JKZ, ‘Jiukengzao’; XX1, ‘Xiaoxiang 1’; FDDB, ‘Fuding Dabaicha’; ZHDB, ‘Zhenghe Dabaicha’; MZ, ‘Mei Zhan’; FJSX, ‘Fujian Shuixian’; PYTZ, ‘Pingyang Tezao’.

#### 3.2.2 Physicochemical properties of PYHT tea made from different cultivars

Color difference of the dry tea and infusion prepared from different cultivars was measured and summarized in Table S11. The L*, a*, b* and ΔE values of dry tea ranged from 56.32 to 61.03, from 0.71 to 1.59, from 11.25 to 18.31, and from 3.21 to 6.12; while those of the infusion ranged from 85.81 to 88.99, from −2.44 to 0.09, from 16.36 to 33.08, and from 5.74 to 15.58, respectively. The variation of color difference in PYHT teas made from the shoots of various cultivars seemed bigger than that of different SY parameters, indicating the cultivar would significantly influence the color of the dry tea and infusion besides the processing technology. According to the analysis of the pigments (Table S12), although the contents of the chlorophylls and pheophytins varied in the samples prepared from the shoots of different cultivars, the low chlorophylls but high pheophytins were observed which was consistent with previous observation under different SY conditions. In addition, similar profile of the carotenoids was observed in samples except the cultivars with yellowish green shoots, such as PYTZ, ZN805, ZHDB and XX1, implying that content of carotenoids was mainly subjected to the cultivars instead of the process technology.

The taste-related compounds of samples made from 15 cultivars were measured and presented in Table S13. The content of polyphenols and free amino acids ranged from 18.12 % to 25.17 %, and from 3.77 % to 6.27 %, resulting in TPs/FAA from 3.60 to 5.97. The profile of catechins in different samples was similar to polyphenols, and level of caffeine ranged from 2.42 % to 4.43 %. It was clear that the contents of the taste-related compounds were significantly influenced by the cultivar besides the process parameters. As presented, the levels of these chemical indexes varied and was potentially responsible for the difference in the color and taste of YT processed from the shoots of different cultivars.

The manufacture suitability of tea cultivars was mainly associated with volatile compounds besides the taste-related compounds (Feng et al., 2024). A total of 65 volatiles were identified by GC/MS from the PYHT teas prepared from the shoots of different cultivars (Table S14), including 25 alcohols, 12 aldehydes, 12 ketones, 11 esters, 2 alkenes, 2 heterocycles, 1 oxime. Among them, alcohols and aldehydes were the main components, accounting for more than 70 % of the total amount. There were 26 volatiles co-owned in these 15 different PYHT teas, and their ROAV values were speculated (Table S15). Relative high ROAV values of 2,4-dimethyl benzaldehyde (546.25–926.58, with bitter almond flavor note), 1-octanol (335.14–1048.54, with green, citrus, fatty, coconut-like notes), *β*-ionone (142.15–3250.39, with violet-like notes), linalool (138.52–593.55, with floral, sweet, grape-like, woody notes), and 2-methyl butyraldehyde (41.27–299.89, with malty note) were observed. Following the optimal sealed yellowing parameters, the tea made from different cultivars also displayed various aroma types as previous, such as faint scent, fragrant and sweet scent. To visualize the contributions of the non-volatile and volatile compounds, a heat map was conducted to determine the concentration distributions of the key compounds (Fig. 6b). Results showed that 15 samples other than PYTZ could be clustered into 2 groups, 5 samples of group 1 were found owned lower content of chlorophylls, pheophytins, lutein and carotenoids, as well as higher content of TPs, caffeine, C, GCG, EGC, galloylated catechins, total catechins, geraniol and benzyl acetate, while a contrary trend of chemical distribution was found in other 9 samples of group 2. Owing to the higher content of pheophytins, *β*-carotene, lutein, total carotenoids, 2-methylbutyraldehyde, hexanal and nonanal, as well as the lower content of TPs, galloylated catechins, total catechins, caffeine, 1-pentanol, 1-hexanol, 1-heptanol and hotrienol, the represent samples of group 2, especially ZC108, ZN801, JKZ and 5–21, exhibited the characteristic of “sweet and cooked” aroma and “sweetish mellow” taste. These findings also confirmed the physio-chemical attributes related to aroma and taste qualities of PYHT teas previously observed in orthogonal design. Meanwhile, the above results also indicated that the difference in the phytochemical content of YT might not only be caused by the differential processing parameters, but also ascribed to the cultivars to a certain extent.

## 4 Discussion

### 4.1 Formation mechanism of PYHT tea quality during sealed yellowing

**Fig. 7:**
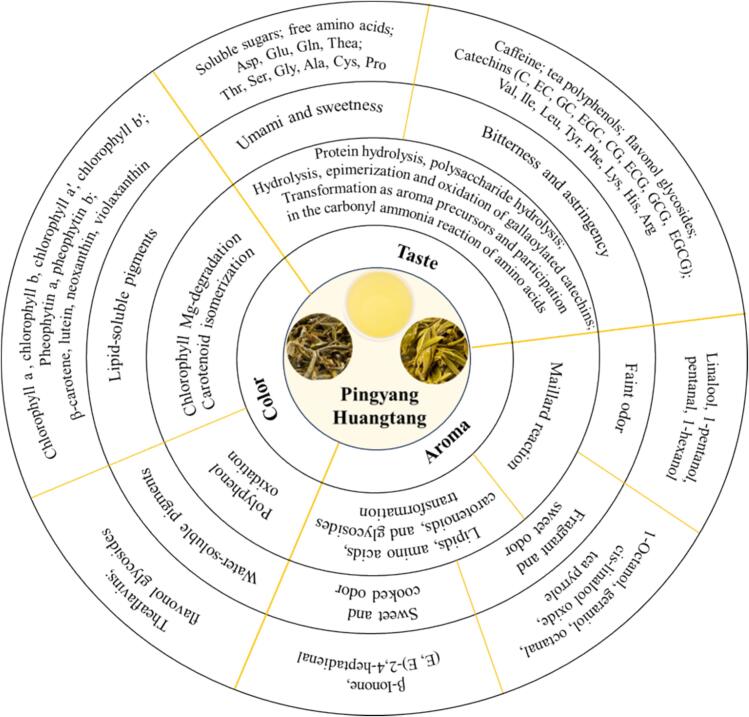
Color, aroma, and taste quality formation mechanism of PYHT tea. Asp, aspartic acid; Glu, glutamic acid; Gln, glutamine; Thea, theanine; Thr, threonine; Ser, serine; Gly, glycine; Ala, alanine; Cys, cysteine; Pro, proline; Val, valine; Ile, isoleucine; Leu, leucine; Tyr, tyrosine; Phe, phenylalanine; Lys, lysine; His, histidine; Arg, arginine.

The color, aroma, and taste quality formation mechanism of PYHT tea was speculated accordingly and summarized in Fig. 7. The color formation of dry tea and infused leaves was mainly attributed to variation of the lipid-soluble pigments, especially the chlorophylls and carotenoids, as well as their derivatives. The temperature was the key factors of chlorophylls degradation during sealed yellowing, high temperature would lead to a large amount of pheophytin formation, resulting in the decrease of greenness and lightness (Feng, et al., 2024). During the processing of sealed yellowing, carotenoids undergone partial isomerization and oxidative reactions, but within a small range, leading to a large number of yellow chemicals remaining (Shi et al., 2023). It was clear that dramatically destroyed chlorophylls but largely preserved carotenoids were the chemical base for “yellow” of dry tea and infused leaves. The color of PYHT tea infusion was probably due to newly synthesized water-soluble pigments such as theaflavins and catechin dimers (Tanaka et al., 2022) which was reflected by the decreased level of catechins during sealed yellowing. According to the sensory evaluation, bitterness and astringency, umami, sweetness were the main taste attributes of PYHT, which might be related to catechins, flavonols and their glycosides, caffeine, soluble sugars and free amino acids (Fan et al., 2022; Sheng et al., 2024). Sealed yellowing at low temperature and relative humidity would be beneficial for maintenance of the green tea-aroma attributes but increased hydrolysis of the polymers such as proteins and polysaccharides, thus leading to obtain the PYHT with faint scent aroma and fresh umami taste unable to meet the typical characteristics of yellow tea; however, sealed yellowing at high temperature would be beneficial for transformation of the ingredients, especially, oxidation and degradation of the TPs, hydrolysis of proteins and polysaccharides as well as the subsequent maillard reaction (Ho, Zheng, & Li, 2015), volatile release through breakdown of the glycoside precursors and coupled oxidation of amino acids as well as unavoidable escape through evaporation (Yin et al., 2023), finally, leading to the decreased TPs (with a relatively big range) and FAA (with a relatively small amplitude) as well as the newly-balanced volatiles. These would be helpful for developing the mellow taste as well as sweet and cooked aroma able to meet the typical quality of yellow tea.

### 4.2 The processing technique determines the lower limit of the quality of yellow tea

Same fresh tea leaves could be manufactured into green tea, white tea, yellow tea, oolong tea, dark tea, and black tea when different processing techniques were adopted (Wang et al., 2019). Furthermore, parameter variation of every process step, sometimes even just minor adjustments, could significantly affect product quality. According to our result of the orthogonal optimization design, the temperature and RH of the sealed yellowing would significantly influence total quality of the PYHT, which was in line with many previous reports on Huoshan Huangya tea (Wei et al., 2020; Wei, et al., 2023) and other yellow tea (Wang, Feng, Min, Yin, & Jiang, 2024), but inconsistent with a previously published paper in which temperature and humidity had no significant effects on the PYHT quality when the sealed yellowing was carried out at 25–35 °C and 70–90 % RH (Dong et al., 2023). Obviously, the range of the temperature (40–60 °C) and RH (50–90 %) in this study was remarkably different with the report, this might be the key to the inconsistent result of the two studies. Recently, parameter optimization of the number of yellowing and water content of dhool (Wei et al., 2020; Wei et al., 2021), total yellowing duration (Fan et al., 2022), temperature and relative humidity (Wang, Feng, Min, Yin, & Jiang, 2024; Wei et al., 2023) had been carried out for improving the quality of the different yellow tea. For the Huoshan Huangya tea, increasing the yellowing temperature from 20 °C to 34 °C, increasing the RH from 55 % to 67 %, and reducing the yellowing duration from 48 h to 16 h, would lead to a 40.5 % and 43.2 % increase in the yellowness and sweetness (Wei et al., 2023), and under the optimized condition, some key odorants including dimethyl sulfide, 3-methylbutanal, *β*-ionone, *β*-damascenone, geraniol, phenylacetaldehyde, and linalool would be at appropriate concentrations (Wei et al., 2024). According to our study, high quality PYHT could be achieved when sealed yellowing was performed at 60 °C, 70 % RH for 10 h with 2 ventilation operation. Under this yellowing condition, the PYHT tea would exhibit the brilliant bright infusion, sweet and cooked aroma, and mellow taste, with coordinated volatiles and suitable TPs/FAA. Obviously, these parameters were different with the reported ones obtained from Huoshan Huangya tea. These might reflect differences in quality control between two regional yellow teas.

### 4.3 The cultivar defines the upper limit of the quality of yellow tea

Manufacturing suitability of a tea cultivar is highly associated with its phytochemical features, for instance, cultivar suitable for manufacturing the black tea usually possesses higher level of catechins and lower contents of free sugars and amino acids (Li et al., 2020). Different tea germplasms exhibit various accumulation patterns of metabolites because of their diverse genetic background, resulting in disparate manufacturing suitability (Li et al., 2022). Black teas produced in Yunnan Province shared their similar flavor attributes, but their infusion intensities were significantly different because these teas were made from multiple cultivars even through a similar processing protocol (Wang et al., 2017). In practice, ‘Pingyang Tezao’ is commonly used to produce the PYHT tea, and considered as a suitable cultivar for processing the PYHT. Using ‘Pingyang Tezao’ as a control, another 14 tea cultivars were selected to manufacture PYHT under the optimal sealed yellowing condition. Our results revealed that ‘Zhenong 801’, ‘Zhongcha 108’, ‘Jiukengzao’ and ‘5–21’ were the suitable cultivars for manufacturing the PYHT tea, since the teas made from these cultivars exhibited better sensory quality, higher levels of color-related pigments, higher contents of sweet/flowery volatiles but lower contents of green grass-like ones, lower levels of bitter/ astringent compounds. Although it is feasible to provide some framework principles and requirements for manufacturing PYHT, such as described above, it is a tough task to propose very precise suitability indicators. Before scientifically distinguishing the actual impact of process and cultivar on the ingredients of tea (Li et al., 2020; Mozumder, Lee, & Hong, 2025), screening out the suitable cultivars through sensory evaluation is still an effective method.

## 5 Conclusions

To summarize, the effects of different sealed yellowing parameters on the quality of PYHT were comprehensively investigated through an orthogonal experiment design, as well as the manufacturing suitability of various tea cultivars for the PYHT tea was systematically evaluated under the optimized yellowing condition. Among the tested three factors, total sensory score of PYHT tea was mainly subjected to the temperature and RH of the sealed yellowing although ventilation frequency had slight effect on the appearance, infusion, aroma and taste scores. The optimal sealed yellowing should be performed at 60 °C, 70 % RH for 10 h with ventilation 2 times. During sealed yellowing, dramatically decreased chlorophylls but a slightly changed carotenoids resulted in the yellowed appearance and infused leaves of the PYHT tea, while a slight oxidation of polyphenols might be responsible for the yellowed infusion. The mellow taste of PYHT was closely related to the decrease in polyphenols, especially the galloylated catechins, and TPs/FAA after sealed yellowing treatment. “Faint scent”, “fragrant and sweet scent” and “sweet and cooked scent”, the typical aroma attributes of the PYHT, were mainly dependent on the content of linalool, 1-pentanol, geraniol, pentanal, (E, E)-2,4-heptadienal, *β*-ionone, 1-octanol, although many volatiles fluctuated during sealed yellowing. According to sensory evaluation and physio-chemical measurement, ‘Zhenong 801’, ‘Zhongcha 108’, ‘Jiukengzao’ and ‘5–21’ were recommended as the suitable cultivars for manufacturing the PYHT tea although the total sensory score of 10 in 14 tested cultivars was higher than that of PYTZ which was commonly used to produce the PYHT in practice. The processing technique determines the lower limit of the quality of yellow tea, but cultivar defines the upper limit of the quality of yellow tea. In the future, it is necessary to strengthen the optimization and integration of the upstream and downstream steps of SY, and carry out research on the breeding of new cultivars especially suitable for producing PYHT. These will lay the foundation for the process standardization and cultivar specialization of PYHT industry.

## CRediT authorship contribution statement

**Shanshan Wu:** Writing – original draft, Visualization, Validation, Software, Investigation. **Dingwu Zhang:** Writing – review & editing, Investigation, Formal analysis. **Siyi Hu:** Writing – original draft, Validation, Methodology, Investigation. **Cunyu Li:** Methodology. **Zhanbo Dong:** Resources, Data curation. **Yurong Hu:** Formal analysis, Data curation. **Fangyuan Fan:** Validation. **Jianhui Ye:** Supervision. **Xinqiang Zheng:** Resources. **Yuerong Liang:** Supervision. **Liaoyuan Yu:** Funding acquisition, Conceptualization. **Jianliang Lu:** Writing – review & editing, Project administration, Funding acquisition, Conceptualization.

## Funding sources

This work was financially supported by the Zhejiang Province Agricultural Major Technology Collaborative Promotion Project (2022XTTGCY01-0), the 10.13039/501100001809National Natural Science Foundation of China (No. 32272763) and Promotion Project of Science and Technology Strengthening Agriculture Industry in Pingyang County (2023PY II 05).

## Declaration of competing interest

The authors declare that they have no known competing financial interests or personal relationships that could have appeared to influence the work reported in this paper.

## Data Availability

Data will be made available on request.

## Appendix A Supplementary data

Supplementary material Image 1
