# Supplementary material for: Optimization of the sealed yellowing parameters and suitability evaluation of the different cultivars for manufacturing the Pingyang Huangtang tea
Source: Food Chem X. 2025 Jun 3;28:102615. doi: 10.1016/j.fochx.2025.102615 (PMC12173723; doi:10.1016/j.fochx.2025.102615)
Supplement: Supplementary file 1 — Supplementary material [file mmc1.docx]

**Optimization of the sealed yellowing parameters and suitability evaluation of the different cultivars for manufacturing the Pingyang Huangtang tea**

Shanshan Wu^a,1^, Dingwu Zhang^b,1^, Siyi Hu^a^, Cunyu Li^a^, Zhanbo Dong^c^, Yurong Hu^b^, Fangyuan Fan^a^, Jianhui Ye^a^, Xinqiang Zheng^a^, Yuerong Liang^a^, Liaoyuan Yu^d,**^, Jianliang Lu^a,*^

^a^Zhejiang University Tea Research Institute, Hangzhou 310058, China

^b^KangShi (Shanghai) Food Science and Technology Co., Ltd.

^c^Wanquan Town People's Government, Pingyang County, Wenzhou 325409, China

^d^Zhejiang Agricultural Technology Extension Center, Hangzhou 310020, China

^1^the authors who contributed equally to this work

⁎ Corresponding author at: 866 Yuhangtang Road, Hangzhou, Zhejiang 310058, China, Email: jllu@zju.edu.cn.

⁎⁎ Corresponding author at: 131 North Qiutao Road, Hangzhou, Zhejiang 310020, China, Email: ylytea@163.com.

**Supplementary captions:**

Table S1. Combination treatments according to orthogonal design L9 (3^3^).

Table S2. Detailed information of the Pingyang Huangtang tea samples in this study.

Table S3. Effect of sealed yellowing factors and levels on the scores of sensory evaluations.

Table S4. Effect of sealed yellowing parameters on total sensory score of the PYHT tea.

Table S5. Effect of the sealed yellowing factors on color differences.

Table S6. Effect of the sealed yellowing factors on the contents of the pigments and their derivatives (μg/g).

Table S7. Effect of the sealed yellowing factors on taste-related components.

Table S8. Relative content of the volatiles in PYHT tea manufactured under different sealed yellowing conditions (μg/g).

Table S9. Total level of each category of volatiles in PYHT tea manufactured under different sealed yellowing conditions (μg/g).

Table S10. Effect of sealed yellowing parameters on the total level of each kind of volatiles (μg/g).

Table S11. Color difference of PYHT tea prepared from the shoots of different tea cultivars.

Table S12. Pigments and their derivatives in PYHT tea prepared from the shoots of different cultivars (μg/g).

Table S13. Taste-related components of PYHT tea prepared from the shoots of different tea cultivars.

Table S14. Relative contents of the volatiles in PYHT tea prepared from the shoots of different cultivars (μg/g).

Table S15. Relative odor activity values of co-owned volatiles screened out from PYHT tea prepared from the shoots of different cultivars.

**Table S1.** Combination treatments for the sealed yellowing factors according to orthogonal design L9 (3^3^).

| Treatments | Temperature (A, ℃) | Relative humidity (B, %) | Ventilation frequency (C) |
| --- | --- | --- | --- |
| YT1 | A1 (40) | B1 (50) | C1 (10) |
| YT2 | A2 (50) | B1 (50) | C2 (6) |
| YT3 | A3 (60) | B1 (50) | C3 (2) |
| YT4 | A1 (40) | B2 (70) | C2 (6) |
| YT5 | A2 (50) | B2 (70) | C3 (2) |
| YT6 | A3 (60) | B2 (70) | C1 (10) |
| YT7 | A1 (40) | B3 (90) | C3 (2) |
| YT8 | A2 (50) | B3 (90) | C1 (10) |
| YT9 | A3 (60) | B3 (90) | C2 (6) |

Note: Water content of the rolled leaves was around 45%, and duration of the sealed yellowing was set to 10h. Data in the brackets were the actual levels of the factor.

**Table S2.** Detailed information of the Pingyang Huangtang tea samples in this study.

| Code | Abbreviation | Description of tea sample | Sealed yellowing condition |
| --- | --- | --- | --- |
| **Yellow tea samples from orthogonal experiment (n=27, only one repetition was displayed)** | | | |
| 1 | YT1 | Treatment combination 1 of sealed yellowing | 40℃ / 50% / 10 |
| 2 | YT2 | Treatment combination 2 of sealed yellowing | 50℃ / 50% / 6 |
| 3 | YT3 | Treatment combination 3 of sealed yellowing | 60℃ / 50% / 2 |
| 4 | YT4 | Treatment combination 4 of sealed yellowing | 40℃ / 70% / 6 |
| 5 | YT5 | Treatment combination 5 of sealed yellowing | 50℃ / 70% / 2 |
| 6 | YT6 | Treatment combination 6 of sealed yellowing | 60℃ / 70% / 10 |
| 7 | YT7 | Treatment combination 7 of sealed yellowing | 40℃ / 90% / 2 |
| 8 | YT8 | Treatment combination 8 of sealed yellowing | 50℃ / 90% / 10 |
| 9 | YT9 | Treatment combination 9 of sealed yellowing | 60℃ / 90% / 6 |
| 10 | GT | Green tea (control) | - |
| **Tea samples of manufacturing suitability experiment (n=45, only one repetition was displayed)** | | | |
| 1 | ZC108 | Tea made from the shoots of 'Zhongcha 108' | 60℃ /70% / 2 |
| 2 | ZN805 | Tea made from the shoots of 'Zhenong 805' | 60℃ /70% / 2 |
| 3 | ZN801 | Tea made from the shoots of 'Zhenong 801' | 60℃ /70% / 2 |
| 4 | ZN901 | Tea made from the shoots of 'Zhenong 901' | 60℃ /70% / 2 |
| 5 | ZN301 | Tea made from the shoots of 'Zhenong 301' | 60℃ /70% / 2 |
| 6 | ZN302 | Tea made from the shoots of 'Zhenong 302' | 60℃ /70% / 2 |
| 7 | JKZ | Tea made from the shoots of 'Jiukengzao' | 60℃ /70% / 2 |
| 8 | 5-21 | Tea made from the shoots of '5-21' | 60℃ /70% / 2 |
| 9 | 1-35 | Tea made from the shoots of '1-35' | 60℃ /70% / 2 |
| 10 | XX1 | Tea made from the shoots of 'Xiaoxiang 1' | 60℃ /70% / 2 |
| 11 | FDDB | Tea made from the shoots of 'Fuding Dabaicha' | 60℃ /70% / 2 |
| 12 | ZHDB | Tea made from the shoots of 'Zhenghe Dabaicha' | 60℃ /70% / 2 |
| 13 | MZ | Tea made from the shoots of 'Mei Zhan' | 60℃ /70% / 2 |
| 14 | FJSX | Tea made from the shoots of 'Fujian Shuixian' | 60℃ /70% / 2 |
| 15 | PYTZ | Tea made from the shoots of 'Pingyang Tezao' | 60℃ /70% / 2 |

Note: Sealed yellowing condition: temperature/relative humidity/ ventilation frequency.

**Table S3.** Effect of sealed yellowing factors and levels on the scores of sensory evaluations.

| Items | Level | Temperature  (A) | Relative humidity  (B) | Ventilation frequency  (C) |
| --- | --- | --- | --- | --- |
| Appearance score | 1 | 89.67 | 90.00 | 89.33 |
|  | 2 | 90.00 | 89.67 | 89.33 |
|  | 3 | 89.33 | 89.33 | 90.33 |
|  | Optimum | A2 | B1 | C3 |
|  | Range | 0.67 | 0.67 | 1.00 |
|  | Contribution rate (%) | 0.74 | 0.74 | 1.12 |
| Infusion color score | 1 | 86.67 | 87.67 | 89.00 |
|  | 2 | 88.67 | 90.67 | 88.33 |
|  | 3 | 91.00 | 88.00 | 89.00 |
|  | Optimum | A3 | B2 | C1/C3 |
|  | Range | 4.33 | 3.00 | 0.67 |
|  | Contribution rate (%) | 4.88 | 3.38 | 0.75 |
| Aroma score | 1 | 87.67 | 89.33 | 89.67 |
|  | 2 | 89.00 | 90.00 | 89.33 |
|  | 3 | 91.00 | 88.33 | 88.67 |
|  | Optimum | A3 | B2 | C1 |
|  | Range | 3.33 | 1.67 | 1.00 |
|  | Contribution rate (%) | 3.74 | 1.87 | 1.12 |
| Taste score | 1 | 86.67 | 88.00 | 88.33 |
|  | 2 | 88.67 | 88.00 | 88.67 |
|  | 3 | 91.00 | 90.33 | 89.33 |
|  | Optimum | A3 | B3 | C3 |
|  | Range | 4.33 | 2.33 | 1.00 |
|  | Contribution rate (%) | 4.88 | 2.63 | 1.13 |
| Infused leaf score | 1 | 85.00 | 87.00 | 87.00 |
|  | 2 | 87.00 | 87.00 | 87.00 |
|  | 3 | 89.00 | 87.00 | 87.00 |
|  | Optimum | A3 | - | - |
|  | Range | 60℃ | - | - |
|  | Contribution rate (%) | 4.60 | 0.00 | 0.00 |
| Total score | 1 | 87.54 | 88.71 | 88.98 |
|  | 2 | 88.86 | 89.16 | 88.97 |
|  | 3 | 90.56 | 89.09 | 89.01 |
|  | Optimum | A3 | B2 | C3 |
|  | Range | 3.02 | 0.46 | 0.03 |
|  | Contribution rate (%) | 3.39 | 0.52 | 0.03 |

Note: Contribution rate (%) = range / mean × 100.

**Table S4.** Effect of sealed yellowing parameters on total sensory score of the PYHT tea.

| Source of variance | Sum of squares | df | Mean square | F | *p* value |
| --- | --- | --- | --- | --- | --- |
| Intercept | 213804.71 | 1 | 213804.71 | 2068892.40 | 0.000 |
| Temperature | 41.32 | 2 | 20.66 | 199.90 | 0.000 |
| Relative humidity | 1.09 | 2 | 0.55 | 5.27 | 0.014 |
| Ventilation frequency | 0.01 | 2 | 0.00 | 0.03 | 0.975 |
| Residual | 2.07 | 20 | 0.10 |  |  |

**Table S5.** Effect of the sealed yellowing factors on color differences.

| Treatments | Color difference of dry tea | | | |  | Color difference of tea infusion | | | |
| --- | --- | --- | --- | --- | --- | --- | --- | --- | --- |
|  | L* | a* | b* | ΔE |  | L* | a* | b* | ΔE |
| A1 | 62.88 | 0.30 | 14.89 | 3.98 |  | 95.95 | -5.46 | 19.31 | 2.60 |
| A2 | 61.84 | 1.63 | 14.01 | 5.40 |  | 93.65 | -2.10 | 26.34 | 9.87 |
| A3 | 61.06 | 2.56 | 13.46 | 6.41 |  | 91.88 | 0.16 | 34.73 | 21.86 |
| B1 | 62.56 | 1.32 | 14.00 | 5.05 |  | 93.90 | -2.29 | 26.47 | 11.81 |
| B2 | 62.09 | 1.63 | 14.06 | 5.28 |  | 93.37 | -1.90 | 28.71 | 13.32 |
| B3 | 61.14 | 1.54 | 14.29 | 5.46 |  | 94.22 | -3.22 | 25.20 | 9.20 |
| C1 | 61.83 | 1.24 | 14.03 | 4.95 |  | 94.09 | -2.78 | 25.53 | 9.85 |
| C2 | 61.89 | 1.41 | 14.03 | 5.31 |  | 94.16 | -2.79 | 25.04 | 10.22 |
| C3 | 62.07 | 1.84 | 14.30 | 5.53 |  | 93.24 | -1.84 | 29.81 | 14.25 |
| A-CR% | 2.93 | 150.78 | 10.13 | 46.10 |  | 4.34 | -227.77 | 57.55 | 168.34 |
| B-CR% | 2.29 | 20.94 | 2.05 | 7.66 |  | 0.91 | -53.20 | 13.10 | 36.01 |
| C-CR% | 0.39 | 40.31 | 1.94 | 10.89 |  | 0.98 | -38.48 | 17.78 | 38.46 |

Note: CR%, contribution rate (%) = range / mean × 100.

**Table S6**. Effect of the sealed yellowing factors on the contents of the pigments and their derivatives (μg/g).

| Treat-  ments | Chlorophylls | | | | |  | Pheophytins | | |  | Carotenoids | | | | |
| --- | --- | --- | --- | --- | --- | --- | --- | --- | --- | --- | --- | --- | --- | --- | --- |
|  | Chl a | Chl a' | Chl b | Chl b' | Chls |  | Py a | Py b | Pys |  | Car | Neo | Vio | Lut | Caros |
| A1 | 12.81 | 5.22 | 37.97 | 12.18 | 68.17 |  | 593.51 | 175.53 | 769.04 |  | 132.47 | 6.22 | 1.89 | 150.97 | 291.54 |
| A2 | 1.64 | 2.62 | 11.86 | 4.20 | 20.32 |  | 786.14 | 177.32 | 963.46 |  | 119.20 | 3.67 | 1.55 | 136.14 | 260.56 |
| A3 | 1.76 | 2.67 | 3.24 | 1.49 | 9.16 |  | 903.06 | 184.45 | 1087.51 |  | 114.00 | 2.82 | 1.36 | 135.54 | 253.71 |
| B1 | 7.09 | 1.97 | 17.71 | 5.94 | 32.71 |  | 600.52 | 141.98 | 742.50 |  | 102.58 | 4.36 | 1.60 | 123.01 | 231.55 |
| B2 | 4.06 | 3.51 | 14.02 | 4.67 | 26.27 |  | 761.64 | 172.26 | 933.90 |  | 112.18 | 3.98 | 1.49 | 133.49 | 251.14 |
| B3 | 5.05 | 5.02 | 21.34 | 7.26 | 38.68 |  | 920.55 | 223.06 | 1143.61 |  | 150.90 | 4.36 | 1.71 | 166.15 | 323.12 |
| C1 | 6.80 | 4.64 | 18.96 | 6.39 | 36.79 |  | 742.58 | 177.06 | 919.64 |  | 121.39 | 4.39 | 1.75 | 141.54 | 269.06 |
| C2 | 4.28 | 3.21 | 16.78 | 5.69 | 29.95 |  | 766.91 | 177.09 | 944.00 |  | 122.96 | 4.18 | 1.53 | 140.51 | 269.19 |
| C3 | 5.12 | 2.66 | 17.34 | 5.78 | 30.91 |  | 773.21 | 183.15 | 956.36 |  | 121.31 | 4.13 | 1.52 | 140.60 | 267.56 |
| A-CR% | 206.81 | 74.24 | 196.28 | 179.59 | 181.30 |  | 40.68 | 4.98 | 33.88 |  | 15.15 | 80.31 | 33.17 | 10.95 | 14.08 |
| B-CR% | 56.10 | 87.18 | 41.40 | 43.44 | 38.13 |  | 42.06 | 45.27 | 42.67 |  | 39.65 | 9.13 | 13.56 | 30.62 | 34.09 |
| C-CR% | 46.78 | 56.35 | 12.32 | 11.70 | 21.00 |  | 4.03 | 3.40 | 3.91 |  | 1.36 | 6.22 | 14.19 | 0.73 | 0.61 |

Note: CR%, contribution rate (%) = range / mean × 100; Chl a, chlorophyll a; Chl a', epimer of chlorophyll a; Chl b, chlorophyll b; Chl b', epimer of chlorophyll b; Chls, total chlorophylls; Py a, pheophytin a; Py b, pheophytin b; Pys, total pheophytins; Car, β-carotene; Neo, neoxanthin; Vio, violaxanthin; Lut, lutein; Caros, total carotenoids. The content of Chl a' and Chl b' was quantified using the Chl a and Chl b as the reference compounds, respectively.

**Table S7.** Effect of the sealed yellowing factors on taste-related components.

| Treatments | TPs (%) | FAA (%) | TPs/FAA | Total catechins (%) | Galloylated  catechins (%) | Non-galloylated  catechins (%) | Caffeine (%) |
| --- | --- | --- | --- | --- | --- | --- | --- |
|  |  |  |  |  |  |  |  |
| A1 | 18.65 | 4.21 | 4.46 | 11.75 | 7.08 | 4.67 | 1.85 |
| A2 | 17.95 | 4.17 | 4.33 | 12.02 | 6.93 | 5.09 | 1.91 |
| A3 | 17.03 | 4.07 | 4.20 | 10.85 | 6.32 | 4.54 | 1.86 |
| B1 | 17.70 | 4.49 | 3.95 | 11.59 | 6.78 | 4.81 | 1.96 |
| B2 | 19.55 | 3.91 | 5.01 | 11.49 | 6.68 | 4.81 | 1.87 |
| B3 | 16.38 | 4.06 | 4.03 | 11.54 | 6.87 | 4.67 | 1.80 |
| C1 | 17.84 | 4.13 | 4.34 | 11.64 | 6.80 | 4.84 | 1.84 |
| C2 | 18.04 | 4.21 | 4.31 | 11.85 | 6.98 | 4.87 | 1.92 |
| C3 | 17.75 | 4.12 | 4.33 | 11.13 | 6.55 | 4.58 | 1.85 |
| A-CR% | 9.04 | 3.45 | 5.93 | 10.14 | 11.27 | 11.55 | 3.20 |
| B-CR% | 17.73 | 13.97 | 24.56 | 0.92 | 2.80 | 3.08 | 8.54 |
| C-CR% | 1.64 | 2.33 | 0.69 | 6.18 | 6.25 | 6.23 | 4.27 |

Note: CR%, contribution rate (%) = range / mean × 100; TPs, total polyphenols; FAA, free amino acids.

**Table S8.** Relative content of the volatiles in PYHT tea manufactured under different sealed yellowing conditions (μg/g).

| Volatiles | RI | YT1 | YT2 | YT3 | YT4 | YT5 | YT6 | YT7 | YT8 | YT9 |
| --- | --- | --- | --- | --- | --- | --- | --- | --- | --- | --- |
| Dimethyl sulfide | 713 | 5.08±0.70a | 3.81±0.15bc | 2.85±0.64cde | 4.52±0.69ab | 3.76±0.37bcd | 2.65±0.33e | 3.21±1.08cde | 3.05±0.69cde | 2.73±0.37de |
| 2-Methylpropionaldehyde | 769 | 0.39±0.23ab | 0.30±0.08abc | 0.15±0.06d | - | 0.40±0.02a | 0.21±0.11cd | 0.36±0.07ab | 0.43±0.03a | 0.26±0.06bcd |
| Ethyl acetate | 847 | 0.94±0.16a | 0.53±0.18b | 0.52±0.18b | 0.63±0.15b | 0.47±0.10b | 0.50±0.07b | 0.63±0.11b | 0.62±0.16b | 0.40±0.19b |
| 2-Butanone | 861 | 0.41±0.28 | - | - | - | - | - | - | - | - |
| 2-Methylbutyraldehyde | 873 | 2.50±0.50ab | 2.14±0.35bcd | 1.74±0.29cd | 2.45±0.35abc | 2.89±0.48a | 1.52±0.41d | 2.23±0.32abcd | 2.96±0.63a | 1.90±0.47bcd |
| 3-Methyl-1-butanol | 925 | 0.76±0.01a | 0.29±0.25b | - | - | 0.55±0.16ab | - | - | - | - |
| Pentanal | 957 | 5.77±0.78a | 3.11±0.56cd | 2.65±0.40cde | 4.60±0.54b | 3.67±0.79bc | 2.47±0.51de | 4.64±1.14ab | 3.16±0.64cd | 1.85±0.30e |
| 1-Penten-3-one | 1019 | 0.60±0.06cd | 0.66±0.13bcd | 0.53±0.04d | 0.83±0.08b | 0.73±0.06bcd | 0.64±0.06bcd | 1.38±0.28a | 1.34±0.07a | 0.80±0.17bc |
| 2-Butenal | 1046 | - | - | - | 0.05±0.02a | 0.06±0.02a | - | 0.06±0.01a | 0.06±0.03a | - |
| 2,3-Pentanedione | 1065 | 0.05±0.00cd | 0.05±0.01cd | 0.04±0.01ef | 0.06±0.00bc | 0.05±0.01de | 0.04±0.00f | 0.08±0.01a | 0.06±0.01b | 0.05±0.00def |
| Butyl acetate | 1072 | 0.08±0.01a | 0.05±0.01bc | 0.05±0.00bc | 0.05±0.01bc | - | 0.04±0.00c | 0.04±0.00c | 0.05±0.01b | 0.05±0.01b |
| Hexanal | 1075 | 2.75±0.21bc | 2.50±0.36c | 2.77±0.21bc | 3.09±0.15ab | 3.29±0.39a | 2.54±0.03c | 2.49±0.47c | 2.45±0.26c | 1.93±0.36d |
| 4-Methyl-4-penten-2-one | 1094 | 0.16±0.01a | 0.12±0.02b | 0.08±0.03bcd | 0.08±0.04bcd | 0.08±0.02bcd | 0.06±0.02d | 0.05±0.00d | 0.07±0.01cd | 0.10±0.02bc |
| 4-Methyl-3-penten-2-one | 1134 | 0.97±0.19ab | 0.79±0.23bcd | 0.72±0.08cd | 0.96±0.15ab | 0.92±0.03abc | 0.65±0.04d | 1.05±0.16a | 1.06±0.03a | 0.76±0.18bcd |
| 1-Penten-3-ol | 1137 | 0.21±0.05bc | 0.21±0.00bc | 0.15±0.03c | 0.23±0.05bc | 0.28±0.05b | 0.14±0.05c | 0.27±0.07b | 0.41±0.14a | 0.30±0.11ab |
| β-Myrcene | 1141 | 0.26±0.06c | 0.57±0.13a | 0.44±0.10ab | 0.52±0.11a | 0.53±0.16a | 0.30±0.04bc | 0.46±0.04a | 0.28±0.02c | 0.27±0.02c |
| (E)-2-Hexenal | 1221 | 0.22±0.02bc | 0.14±0.03de | 0.11±0.02e | 0.25±0.02ab | 0.18±0.03cd | 0.11±0.01e | 0.30±0.06a | 0.22±0.04bc | 0.17±0.04cd |
| β-Ocimene | 1246 | 0.16±0.03d | 0.52±0.09a | 0.34±0.15bc | 0.44±0.14ab | 0.30±0.03bcd | 0.19±0.05cd | 0.44±0.12ab | 0.17±0.05d | 0.24±0.02cd |
| 1-Pentanol | 1265 | 1.43±0.26a | 0.89±0.12bc | 0.68±0.20cd | 1.15±0.15b | 0.67±0.08cd | 0.53±0.06d | 1.00±0.19b | 0.58±0.10d | 0.46±0.17d |
| Octanal | 1285 | 0.16±0.03ab | 0.18±0.10a | 0.15±0.04ab | 0.14±0.02ab | 0.14±0.03ab | 0.10±0.02b | 0.10±0.02b | - | - |
| 1-Octen-3-one | 1300 | 0.08±0.01abcd | 0.08±0.04abc | 0.05±0.01cd | 0.11±0.04a | 0.08±0.01abcd | 0.05±0.01bcd | 0.09±0.03ab | 0.09±0.03ab | 0.05±0.00d |
| 2,2,6-Trimethyl-cyclohexanone | 1305 | - | 0.08±0.01bcd | 0.07±0.01cd | 0.10±0.02ab | 0.09±0.01abc | 0.05±0.00d | 0.08±0.02abcd | 0.07±0.02cd | 0.11±0.03a |
| (Z)-2-Heptenal | 1328 | 0.29±0.03ab | 0.27±0.13ab | 0.18±0.03bc | 0.32±0.13a | 0.24±0.04abc | 0.17±0.03bc | 0.32±0.05a | 0.24±0.09abc | 0.13±0.02c |
| 2,3-Octanedione | 1330 | 0.51±0.12a | 0.25±0.02bcde | 0.19±0.02cde | 0.33±0.12b | 0.26±0.10bcde | 0.17±0.06de | 0.32±0.06bc | 0.26±0.09bcd | 0.12±0.03e |
| (Z)-2-Penten-1-ol | 1341 | 0.09±0.05f | 0.17±0.03cd | 0.10±0.01ef | 0.16±0.02cd | 0.17±0.00cd | 0.14±0.03de | 0.23±0.03b | 0.28±0.01a | 0.20±0.05bc |
| 6-Methyl-5-hepten-2-one | 1347 | 0.32±0.05c | 0.35±0.07bc | 0.35±0.07abc | 0.45±0.05a | 0.42±0.02ab | 0.39±0.03abc | 0.43±0.03ab | 0.45±0.08ab | 0.40±0.08abc |
| 1-Hexanol | 1373 | 0.23±0.04a | 0.26±0.09a | 0.23±0.09a | 0.23±0.01a | 0.19±0.01ab | 0.12±0.02b | 0.22±0.04a | 0.13±0.02b | 0.13±0.02b |
| Nonanal | 1387 | 1.08±0.23a | 0.99±0.48a | 0.45±0.09bc | 0.69±0.62ab | 0.61±0.10abc | 0.47±0.09bc | 0.26±0.09bc | 0.18±0.02c | 0.17±0.01c |
| (Z)-3-Hexen-1-ol | 1400 | - | 0.33±0.12abc | 0.27±0.05abcd | - | 0.40±0.18a | 0.20±0.06cd | 0.38±0.07ab | 0.24±0.10bcd | 0.13±0.04d |
| 3-Octen-2-one | 1411 | - | 0.05±0b | - | - | 0.06±0.01a | - | - | - | - |
| (E)-3-Hexen-1-ol | 1421 | 0.13±0.17c | 0.33±0.12ab | 0.27±0.05abc | 0.14±0.21bc | 0.38±0.13a | 0.22±0.05abc | 0.38±0.07a | 0.24±0.10abc | 0.13±0.04bc |
| (E)-2-Octenal | 1429 | 0.30±0.04de | 0.28±0.04de | 0.35±0.09bcd | 0.41±0.05ab | 0.32±0.06cd | 0.32±0.03cd | 0.48±0.01a | 0.40±0.08abc | 0.22±0.03e |
| Cis-linalol oxide | 1448 | 1.65±0.11b | 1.66±0.19b | 1.22±0.25cd | 1.95±0.17a | 1.44±0.19bc | 0.99±0.19d | 2.14±0.15a | 1.18±0.12cd | 1.07±0.09d |
| 1-Octen-3-ol | 1466 | 0.17±0.01bcd | 0.20±0.04ab | 0.19±0.05abc | 0.21±0.04ab | 0.23±0.06a | 0.14±0.02cd | 0.18±0.02abc | 0.16±0.01bcd | 0.12±0.01d |
| 1-Heptanol | 1472 | 0.13±0.09ab | 0.17±0.03ab | 0.13±0.04ab | 0.23±0.16a | 0.13±0.02ab | 0.13±0.03ab | 0.19±0.11ab | 0.10±0.01b | 0.10±0.02b |
| (E, E)-2,4-Heptadienal | 1475 | 1.07±0.21e | 2.14±0.37c | 1.51±0.15de | 1.74±0.20cd | 1.76±0.12cd | 1.96±0.43cd | 2.64±0.26b | 3.77±0.15a | 2.63±0.35b |
| 2-Ethyl-1-hexanol | 1503 | 2.98±0.44d | 3.51±0.87cd | 4.74±0.49ab | 4.05±0.35bcd | 4.16±0.45bcd | 3.96±0.58bcd | 4.50±0.34abc | 5.46±0.69a | 4.78±1.38ab |
| Benzaldehyde | 1527 | 1.28±0.10 | - | - | - | - | - | - | - | - |
| (E)-2-Nonenal | 1537 | 0.09±0.01b | 0.15±0.02ab | 0.10±0.04b | 0.20±0.04a | 0.10±0.03b | 0.13±0.10ab | 0.16±0.04ab | 0.10±0.04b | - |
| Linalool | 1563 | 4.30±0.18b | 3.16±0.36c | 2.62±0.28cd | 4.05±0.49b | 2.52±0.17d | 1.67±0.17e | 5.93±0.50a | 3.14±0.44c | 2.53±0.28d |
| 1-Octanol | 1573 | 0.72±0.01ab | 0.79±0.15a | 0.63±0.07bc | 0.81±0.06a | 0.62±0.07bc | 0.49±0.03de | 0.72±0.07ab | 0.58±0.05cd | 0.41±0.06e |
| 3,5-Octadien-2-one | 1577 | 0.05±0.01c | 0.10±0.04bc | 0.09±0.02bc | 0.19±0.02a | 0.12±0.05b | 0.08±0.04bc | 0.09±0.01bc | 0.09±0.03bc | 0.06±0.01c |
| Isophorone | 1592 | 0.20±0.01bc | - | 0.64±0.06a | - | - | - | 0.11±0.03cd | 0.06±0.03d | 0.27±0.11b |
| 1-Acetyl-2-methylcyclopentene | 1600 | 0.16±0.01a | - | 0.12±0.01b | - | - | 0.12±0.01b | - | - | - |
| Tea pyrrole | 1613 | 0.12±0.02bc | 0.17±0.02ab | 0.22±0.12a | 0.15±0.03abc | 0.15±0.01abc | 0.08±0.03c | 0.11±0.02bc | 0.10±0.01c | 0.13±0.01bc |
| β-Cyclocitral | 1616 | 0.22±0.01c | 0.28±0.02bc | 0.26±0.04bc | 0.27±0.03bc | 0.27±0.04bc | 0.26±0.04bc | 0.36±0.04a | 0.29±0.06b | 0.37±0.02a |
| Hotrienol | 1626 | 0.12±0.04cd | 0.15±0.03c | 0.11±0.04cd | 0.12±0.02cd | 0.12±0.01cd | 0.09±0.02d | 0.23±0.01a | 0.19±0.01b | 0.19±0.01ab |
| Safranal | 1638 | - | - | 0.31±0.05a | 0.26±0.01ab | 0.28±0.03ab | 0.25±0.04ab | 0.23±0.03b | 0.24±0.02b | 0.27±0.06ab |
| Benzeneacetaldehyde | 1657 | 0.41±0.11d | 0.73±0.23ab | 0.47±0.10cd | 0.53±0.05bcd | 0.53±0.07bcd | 0.45±0.04d | 0.86±0.23a | 0.71±0.02ab | 0.68±0.10abc |
| 1-Nonanol | 1674 | 0.75±0.06a | 0.66±0.00a | 0.34±0.07b | 0.23±0.06bc | 0.36±0.06b | 0.15±0.07c | 0.33±0.17b | 0.26±0.08bc | 0.24±0.12bc |
| α-Terpineol | 1708 | - | - | 0.16±0.02a | - | - | 0.11±0.07a | - | - | 0.17±0.06a |
| Vinyl hexanoate | 1714 | 0.15±0.04b | - | - | 0.18±0.03ab | 0.16±0.03b | 0.18±0.03ab | 0.19±0.02ab | 0.27±0.18a | 0.14±0.02b |
| 2-Ethylbutyric acid | 1712 | - | - | - | 0.40±0.07a | 0.22±0.02b | 0.21±0.04b | 0.38±0.03a | 0.18±0.03b | 0.11±0.06c |
| Linalool oxide (pyranoid) | 1751 | 0.16±0.02bc | 0.34±0.19ab | 0.18±0.04bc | 0.43±0.31a | 0.16±0.03bc | 0.12±0.05c | 0.15±0.01bc | 0.05±0.01c | 0.12±0.07bc |
| 1-Decanol | 1756 | - | 0.11±0.02ab | 0.13±0.09ab | - | 0.19±0.12a | 0.08±0.00b | - | 0.13±0.01ab | - |
| Methyl salicylate | 1779 | 1.38±0.08bcd | 1.59±0.21abc | 1.36±0.38bcd | 1.58±0.18abc | 1.08±0.04cd | 0.91±0.05d | 2.00±0.40a | 1.14±0.13cd | 1.70±0.72ab |
| 2,4-Dimethyl-benzaldehyde | 1818 | 14.41±1.44abc | 12.69±3.18bcd | 10.77±1.78d | 12.20±1.28cd | 11.13±0.92d | 14.82±0.27abc | 14.73±1.13abc | 16.02±0.49a | 15.07±1.91ab |
| Isogeraniol | 1828 | 0.13±0.07a | 0.22±0.09a | - | - | - | - | 0.22±0.15a | - | - |
| α-Ionone | 1855 | - | - | - | - | 0.10±0.01a | - | 0.07±0.01b | 0.07±0.01b | 0.08±0.02b |
| Geraniol | 1870 | 4.87±0.39ab | 4.86±0.52ab | 4.83±0.45ab | 5.24±0.46a | 4.28±0.15bc | 3.69±0.28c | 5.33±0.36a | 4.11±0.40c | 3.69±0.33c |
| Benzyl alcohol | 1890 | 0.21±0.02c | 0.43±0.06ab | 0.52±0.25a | 0.49±0.07a | 0.29±0.01bc | 0.22±0.04c | 0.24±0.05c | 0.24±0.03c | 0.28±0.08bc |
| Phenylethyl alcohol | 1924 | 0.13±0.01e | 0.24±0.02a | 0.19±0.03bc | 0.19±0.05b | 0.17±0.01bcd | 0.15±0.02cde | 0.16±0.01bcde | 0.15±0.02de | 0.17±0.01bcd |
| β-Ionone | 1941 | 0.28±0.06e | 0.28±0.05e | 0.31±0.04cde | 0.29±0.05de | 0.36±0.05bcd | 0.32±0.03bcde | 0.38±0.03bc | 0.39±0.03ab | 0.46±0.03a |
| Jasmone | 1947 | 0.07±0.02c | 0.08±0.00c | - | 0.11±0.01a | - | 0.09±0.01bc | 0.10±0.00ab | 0.07±0.01c | 0.08±0.00c |
| Pentadecanal | 2003 | - | - | 0.12±0.06a | 0.10±0.02a | 0.08±0.00a | - | - | 0.10±0.01a | - |
| Nerolidol | 2064 | 0.57±0.07bc | - | 0.58±0.18bc | 0.94±0.22a | 0.48±0.06c | 0.72±0.03b | 0.43±0.07cd | 0.26±0.04d | - |
| Cedrol | 2116 | 0.25±0.07a | 0.43±0.36a | - | - | - | - | - | - | - |
| Diethyl phthalate | 2387 | - | - | - | - | - | - | 0.71±0.29a | - | - |

Note: The treatment was same as Table S1. Retention index (RI) was calculated from the retention times of the volatiles and a homologous series of n-alkanes (C7-C30). Different letters in a row indicated significant difference at *p*<0.05. -. not detected.

**Table S9.** Total level of each category of volatiles in PYHT tea manufactured under different sealed yellowing conditions (μg/g).

| Treatments | Aldehydes | Alcohols | Ketones | Esters | Acids | Alkenes | Others | Total |
| --- | --- | --- | --- | --- | --- | --- | --- | --- |
| YT1 | 31.07 | 19.96 | 3.71 | 2.54 | 0.00 | 0.58 | 5.08 | 62.94 |
| YT2 | 26.06 | 19.41 | 2.89 | 2.17 | 0.00 | 1.09 | 3.81 | 55.43 |
| YT3 | 22.30 | 18.26 | 3.07 | 1.92 | 0.00 | 0.89 | 2.85 | 49.29 |
| YT4 | 27.47 | 20.85 | 3.50 | 2.44 | 0.40 | 0.96 | 4.52 | 60.14 |
| YT5 | 26.09 | 17.78 | 3.27 | 1.71 | 0.22 | 0.84 | 3.76 | 53.67 |
| YT6 | 25.85 | 14.06 | 2.54 | 1.62 | 0.21 | 0.61 | 2.65 | 47.54 |
| YT7 | 30.34 | 23.22 | 4.24 | 3.57 | 0.38 | 0.90 | 3.21 | 65.86 |
| YT8 | 31.42 | 17.92 | 4.08 | 2.08 | 0.18 | 0.45 | 3.05 | 59.18 |
| YT9 | 25.79 | 15.24 | 3.33 | 2.30 | 0.11 | 0.51 | 2.73 | 50.01 |
| Green tea | 24.48 | 19.67 | 2.44 | 2.96 | 0.00 | 0.60 | 3.94 | 54.08 |

Note: The treatment was same as Table S1, and represented combination of different temperature (40ºC, 50ºC, 60ºC), relative humidity (50%, 70%, 90%) and ventilation frequency (10, 6, 2) during sealed yellowing process.

**Table S10.** Effect of sealed yellowing parameters on the total level of each kind of volatiles (μg/g).

| Treatments | Aldehydes | Alcohols | Ketones | Esters | Acids | Alkenes | Others | Total |
| --- | --- | --- | --- | --- | --- | --- | --- | --- |
| A1 | 29.63 | 21.34 | 3.82 | 2.85 | 0.26 | 0.81 | 4.27 | 62.98 |
| A2 | 27.86 | 18.37 | 3.41 | 1.99 | 0.13 | 0.79 | 3.54 | 56.09 |
| A3 | 24.65 | 15.85 | 2.98 | 1.95 | 0.11 | 0.67 | 2.74 | 48.95 |
| B1 | 26.48 | 19.21 | 3.22 | 2.21 | 0.00 | 0.85 | 3.91 | 55.89 |
| B2 | 26.47 | 17.56 | 3.10 | 1.92 | 0.28 | 0.80 | 3.64 | 53.78 |
| B3 | 29.18 | 18.79 | 3.88 | 2.65 | 0.22 | 0.62 | 3.00 | 58.35 |
| C1 | 29.45 | 17.31 | 3.44 | 2.08 | 0.13 | 0.55 | 3.59 | 56.55 |
| C2 | 26.44 | 18.50 | 3.24 | 2.30 | 0.17 | 0.85 | 3.69 | 55.19 |
| C3 | 26.24 | 19.75 | 3.53 | 2.40 | 0.20 | 0.88 | 3.27 | 56.27 |
| A-CR% | 18.19 | 29.64 | 24.58 | 39.95 | 92.00 | 18.89 | 43.40 | 25.06 |
| B-CR% | 9.91 | 8.89 | 22.92 | 32.14 | 166.00 | 30.75 | 26.06 | 8.15 |
| C-CR% | 11.70 | 13.17 | 8.42 | 14.15 | 42.00 | 43.48 | 11.75 | 2.43 |

Note: CR%, contribution rate (%) = range / mean × 100.

**Table S11.** Color difference of PYHT tea prepared from the shoots of different tea cultivars.

| Cultivars | Color difference of dry tea | | | |  | Color difference of infusion | | | |
| --- | --- | --- | --- | --- | --- | --- | --- | --- | --- |
|  | L* | a* | b* | ΔE |  | L* | a* | b* | ΔE |
| ZC108 | 57.39±0.48f | 1.20±0.05c | 15.48±0.07cd | 6.12±0.25a |  | 88.04±0.02e | -2.03±0.01l | 22.36±0.08f | 8.30±0.01h |
| ZN805 | 58.67±0.40cdef | 1.46±0.04b | 14.18±0.11f | 5.05±0.16abcd |  | 87.36±0.03g | -0.74±0.01d | 23.14±0.07e | 10.00±0.02f |
| ZN801 | 57.66±0.20ef | 1.04±0.06def | 14.93±0.22def | 4.29±0.29cde |  | 88.17±0.02c | -2.44±0.01o | 21.99±0.14g | 7.13±0.09j |
| ZN901 | 58.54±0.63def | 1.13±0.03cd | 15.37±0.44cd | 3.77±0.07de |  | 88.80±0.01b | -1.67±0.01j | 17.30±0.05j | 7.12±0.02j |
| ZN301 | 58.82±1.31cdef | 1.44±0.08b | 16.63±1.10b | 5.37±0.38abc |  | 85.81±0.01k | -0.47±0.00c | 33.08±0.09a | 15.58±0.02a |
| ZN302 | 57.71±0.29ef | 1.04±0.02def | 14.59±0.25def | 5.41±0.08ab |  | 87.35±0.01g | -0.89±0.01f | 24.83±0.07d | 11.67±0.04c |
| JKZ | 58.71±0.46cdef | 1.11±0.04cde | 14.26±0.19ef | 4.84±0.26bcd |  | 87.12±0.01i | 0.09±0.01a | 22.41±0.05f | 11.70±0.02c |
| 5-21 | 58.80±0.16cdef | 0.96±0.02fgh | 14.82±0.27def | 4.50±0.05abcd |  | 87.18±0.01h | -1.08±0.01g | 23.28±0.07e | 10.10±0.01e |
| 1-35 | 59.23±0.90bcde | 0.92±0.02gh | 16.37±0.80bc | 4.12±0.15de |  | 87.05±0.02j | -2.32±0.00n | 27.82±0.08c | 12.13±0.03b |
| XX1 | 60.23±0.20abc | 1.00±0.02efg | 15.18±0.28def | 3.62±0.21e |  | 88.11±0.01d | -0.80±0.01e | 17.02±0.04k | 6.39±0.02l |
| FDDB | 61.03±0.45a | 0.71±0.07i | 18.31±0.09a | 6.02±0.22a |  | 87.19±0.02h | -2.25±0.01m | 27.99±0.09b | 11.17±0.03d |
| ZHDB | 60.70±0.63ab | 0.88±0.06h | 15.30±0.30de | 3.21±0.23e |  | 87.56±0.02f | -1.17±0.01h | 21.16±0.03h | 9.73±0.01g |
| MZ | 59.70±0.49abcd | 1.40±0.07b | 15.41±0.11cd | 3.62±0.26e |  | 88.07±0.03de | -0.42±0.01b | 16.36±0.12l | 7.65±0.02i |
| FJSX | 60.50±0.46ab | 1.11±0.05cde | 17.31±0.06b | 5.44±0.09cde |  | 88.17±0.04c | -1.27±0.01i | 18.20±0.04i | 6.67±0.01k |
| PYTZ | 56.32±1.04g | 1.59±0.06a | 11.25±0.79g | 5.05±0.36abcd |  | 88.99±0.01a | -1.76±0.01k | 17.11±0.02k | 5.74±0.03m |

Note: ZC108, 'Zhongcha 108'; ZN805, 'Zhenong 805'; ZN801, 'Zhenong 801'; ZN901, 'Zhenong 901'; ZN301, 'Zhenong 301'; ZN302, 'Zhenong 302'; JKZ, 'Jiukengzao'; XX1, 'Xiaoxiang 1'; FDDB, 'Fuding Dabaicha'; ZHDB, 'Zhenghe Dabaicha'; MZ, 'Mei Zhan'; FJSX, 'Fujian Shuixian'; PYTZ, 'Pingyang Tezao'. TPs, total polyphenols; FAA, free amino acids. The total color difference (ΔE) was calculated using green tea as control. Different letters in a column indicated significant difference at *p*<0.05.

**Table S12.** Pigments and their derivatives in PYHT tea prepared from the shoots of different cultivars (μg/g).

| Cultivars | Chlorophylls | | | | |  | Pheophytins | | |  | Carotenoids | | | | |
| --- | --- | --- | --- | --- | --- | --- | --- | --- | --- | --- | --- | --- | --- | --- | --- |
|  | Chl a | Chl a' | Chl b | Chl b' | Chls |  | Py a | Py b | Pys |  | Car | Neo | Vio | Lut | Caros |
| ZC108 | 1.98±0.81ab | 2.07±0.86a | 11.47±1.14cd | 4.11±0.22ab | 19.64±0.83ab |  | 584.63±23.97ab | 136.49±10.28a | 721.13±34.22a |  | 125.19±3.72e | 4.03±0.28gh | 1.73±0.06def | 128.22±6.03bc | 259.16±9.60cd |
| ZN805 | 1.30±0.29cd | 0.00±0.00f | 5.37±0.38hi | 1.75±0.24f | 8.42±0.71g |  | 190.46±9.13i | 56.42±10.31i | 246.88±18.97h |  | 79.28±3.67i | 5.21±1.46fg | 1.50±0.6ef | 76.27±7.54h | 162.25±12.64h |
| ZN801 | 1.46±0.22cd | 1.22±0.13bc | 7.01±0.99fg | 2.41±0.4de | 12.10±1.53ef |  | 443.58±42.71e | 95.57±11.23f | 539.15±53.91d |  | 121.61±12.58ef | 6.69±1.26e | 1.75±0.26def | 115.9±12.70e | 245.96±26.7de |
| ZN901 | 1.56±0.30bcd | 0.86±0.19bcde | 9.67±0.75e | 3.21±0.16c | 15.30±1.05d |  | 467.03±5.89e | 116.8±4.61cd | 583.83±5.57c |  | 116.34±1.51f | 3.94±0.14gh | 1.35±0.21f | 119.03±3.54de | 240.66±4.71e |
| ZN301 | 1.19±0.15d | 1.25±0.20b | 13.99±0.77a | 4.29±0.15a | 20.72±1.26a |  | 521.74±8.67d | 118.89±3.28c | 640.63±11.38b |  | 138.10±2.14cd | 8.66±0.35c | 2.21±0.10bcd | 148.06±1.21a | 297.03±0.94b |
| ZN302 | 1.53±0.24bcd | 0.69±0.27de | 10.06±0.56e | 3.11±0.09c | 15.39±0.91d |  | 453.56±10.32e | 105.73±4.16e | 559.29±11.26cd |  | 117.72±3.73f | 6.00±0.84ef | 2.13±0.04bcd | 113.03±3.84ef | 238.88±8.36e |
| JKZ | 1.27±0.13d | 0.53±0.05e | 7.62±0.50f | 2.26±0.17e | 11.68±0.54ef |  | 332.23±11.47g | 74.67±5.45h | 406.90±14.29f |  | 102.50±3.94g | 9.62±0.61c | 2.14±0.47bcd | 105.93±3.74f | 220.20±7.36f |
| 5-21 | 1.45±0.12cd | 0.82±0.06bcde | 11.4±0.53d | 3.38±0.11c | 17.04±0.55c |  | 524.13±13.97d | 124.97±2.05bc | 649.10±16.01b |  | 134.60±2.53d | 8.43±0.21cd | 2.25±0.08bcd | 125.69±1.63cd | 270.97±3.62c |
| 1-35 | 1.34±0.14cd | 0.87±0.10bcde | 12.82±1.06b | 3.85±0.15b | 18.87±1.09b |  | 575.96±35.17bc | 134.19±5.87ab | 710.15±39.21a |  | 149.77±4.57b | 9.39±0.94c | 2.44±0.41abc | 156.95±8.16a | 318.54±13.97a |
| XX1 | 1.24±0.07d | 0.43±0.10ef | 6.90±0.34fg | 2.32±0.15de | 10.89±0.59f |  | 266.28±2.60h | 64.78±3.86hi | 331.06±5.33g |  | 93.90±1.68h | 7.24±1.28de | 2.02±0.57cde | 92.00±4.49g | 195.16±7.88g |
| FDDB | 1.68±0.31bcd | 1.06±0.24bcd | 12.50±0.4bc | 4.10±0.37ab | 19.35±0.73ab |  | 605.97±2.61a | 117.71±5.64c | 723.69±5.35a |  | 143.42±0.61bc | 12.00±1.05b | 2.67±0.21ab | 149.24±5.04a | 307.34±6.88ab |
| ZHDB | 1.20±0.11d | 0.49±0.11e | 4.43±0.62i | 1.67±0.16f | 7.80±0.58g |  | 279.99±12.77h | 66.01±5.02hi | 346.00±16.26g |  | 90.29±0.38h | 9.07±0.29c | 1.80±0.04def | 92.57±2.84g | 193.73±2.33g |
| MZ | 1.78±0.23bc | 0.66±0.24de | 6.16±0.07gh | 2.31±0.10de | 10.90±0.16f |  | 547.85±4.13cd | 106.92±0.21de | 654.77±3.96b |  | 134.55±1.30d | 8.76±0.04c | 2.08±0.27cde | 117.88±1.77de | 263.27±2.76c |
| FJSX | 2.35±0.31a | 0.78±0.29cde | 7.13±0.19fg | 2.64±0.09d | 12.90±0.25e |  | 383.77±9.10f | 85.22±2.87g | 468.98±11.75e |  | 159.31±3.37a | 14.01±0.27a | 3.01±0.18a | 135.9±3.10b | 312.23±6.72ab |
| PYTZ | 1.22±0.14d | 0.52±0.11e | 2.05±0.29j | 0.82±0.11g | 4.61±0.47h |  | 136.07±5.35j | 43.96±2.00j | 180.03±7.35i |  | 42.03±1.47j | 3.71±0.57h | 1.34±0.70f | 34.99±1.00i | 82.07±0.59i |

Note: ZC108, 'Zhongcha 108'; ZN805, 'Zhenong 805'; ZN801, 'Zhenong 801'; ZN901, 'Zhenong 901'; ZN301, 'Zhenong 301'; ZN302, 'Zhenong 302'; JKZ, 'Jiukengzao'; XX1, 'Xiaoxiang 1'; FDDB, 'Fuding Dabaicha'; ZHDB, 'Zhenghe Dabaicha'; MZ, 'Mei Zhan'; FJSX, 'Fujian Shuixian'; PYTZ, 'Pingyang Tezao'; Chla, chlorophyll a; Chla', epimer of chlorophyll a; Chlb, chlorophyll b; Chlb', epimer of chlorophyll b; Chls, total chlorophylls; Pya, pheophytin a; Pyb, pheophytin b; Pys, total pheophytins; Car, β-carotene; Neo, neoxanthin; Vio, violaxanthin; Lut, lutein; Caros, total carotenoids. The content of Chla' and Chlb' was quantified using the Chla and Chlb as the reference compounds, respectively. Different letters in a column indicated significant difference at *p*<0.05.

**Table S13.** Taste-related components of PYHT tea prepared from the shoots of different tea cultivars.

| Cultivars | TPs (%) | FAA (%) | TPs/FAA | Total catechins  (%) | Galloylated  Catechins (%) | Non-galloylated  Catechins (%) | Caffeine (%) |
| --- | --- | --- | --- | --- | --- | --- | --- |
|  |  |  |  |  |  |  |  |
| ZC108 | 18.12±0.61g | 4.89±0.11c | 3.71±0.19fg | 12.79±0.07i | 8.13±0.03h | 4.66±0.02h | 2.73±0.02j |
| ZN805 | 23.20±0.75abc | 3.89±0.05fg | 5.97±0.26a | 14.81±0.25de | 8.99±0.15fg | 5.82±0.05c | 3.49±0.07e |
| ZN801 | 21.73±0.89cd | 4.36±0.11de | 4.98±0.31d | 14.26±0.33fg | 9.02±0.13fg | 5.24±0.14ef | 2.98±0.05h |
| ZN901 | 21.26±1.65cde | 4.21±0.19defg | 5.06±0.38d | 13.73±0.09h | 8.97±0.02g | 4.76±0.05h | 2.42±0.02k |
| ZN301 | 21.90±1.34cd | 3.77±0.08g | 5.81±0.23ab | 14.65±0.18ef | 7.43±0.13i | 7.23±0.02a | 2.83±0.02i |
| ZN302 | 19.38±0.67efg | 3.93±0.03efg | 4.93±0.13d | 13.91±0.32gh | 8.30±0.16h | 5.62±0.11d | 3.10±0.08g |
| JKZ | 22.79±0.87bcd | 4.05±0.16efg | 5.64±0.13abc | 15.47±0.41bc | 9.29±0.23ef | 6.18±0.10b | 3.63±0.12d |
| 5-21 | 24.49±0.63ab | 4.78±0.30c | 5.14±0.21cd | 15.79±0.52b | 10.45±0.32cd | 5.33±0.11e | 3.38±0.12f |
| 1-35 | 20.85±0.63def | 5.55±0.27b | 3.76±0.08efg | 14.32±0.25fg | 8.76±0.12g | 5.57±0.09d | 2.83±0.03i |
| XX1 | 25.17±0.93a | 4.23±0.16def | 5.97±0.43a | 16.64±0.13a | 10.79±0.08b | 5.85±0.03c | 4.43±0.03a |
| FDDB | 21.93±0.25cd | 4.10±0.13efg | 5.35±0.11bcd | 15.50±0.07bc | 9.53±0.04e | 5.96±0.01c | 3.02±0.01gh |
| ZHDB | 24.20±0.92ab | 5.63±0.28b | 4.30±0.13e | 15.63±0.22b | 10.51±0.15bc | 5.13±0.03f | 3.38±0.02f |
| MZ | 24.48±0.95ab | 4.29±0.17def | 5.72±0.44ab | 16.91±0.23a | 11.24±0.13a | 5.68±0.06d | 3.68±0.04d |
| FJSX | 22.56±0.93bcd | 6.27±0.30a | 3.60±0.09g | 15.54±0.10bc | 9.35±0.06e | 6.19±0.02b | 3.93±0.02c |
| PYTZ | 19.16±0.52fg | 4.56±0.21cd | 4.21±0.30ef | 15.14±0.23cd | 10.16±0.18d | 4.97±0.06g | 4.11±0.04b |

Note: ZC108, 'Zhongcha 108'; ZN805, 'Zhenong 805'; ZN801, 'Zhenong 801'; ZN901, 'Zhenong 901'; ZN301, 'Zhenong 301'; ZN302, 'Zhenong 302'; JKZ, 'Jiukengzao'; XX1, 'Xiaoxiang 1'; FDDB, 'Fuding Dabaicha'; ZHDB, 'Zhenghe Dabaicha'; MZ, 'Mei Zhan'; FJSX, 'Fujian Shuixian'; PYTZ, 'Pingyang Tezao'. TPs, total polyphenols; FAA, free amino acids. The total color difference (ΔE) was calculated using green tea as control. Different letters in a column indicated significant difference at *p*<0.05.

**Table S14.** Relative contents of the volatiles in PYHT tea prepared from the shoots of different cultivars (μg/g).

| Volatiles | RI | ZC108 | ZN805 | ZN801 | ZN901 | ZN301 | ZN302 | JKZ | 5-21 | 1-35 | XX1 | FDDB | ZHDB | MZ | FJSX | PYTZ |
| --- | --- | --- | --- | --- | --- | --- | --- | --- | --- | --- | --- | --- | --- | --- | --- | --- |
| Ethyl acetate | 847 | 0.90±0.14cde | 0.49±0.07e | 1.57±0.51abc | 1.33±0.53bcd | 1.81±1.27ab | 1.75±0.18ab | 1.51±0.10abc | 1.36±0.74bcd | 1.68±0.10ab | 0.43±0.02e | 1.46±0.97abcd | 1.47±0.25abcd | 2.10±0.33a | 1.37±0.36abcd | 0.71±0.13de |
| 2-Methylfuran | 850 | 0.10±0.01d | - | - | 0.50±0.08bc | - | 0.53±0.07b | 0.45±0.01c | 0.80±0.13a | - | - | - | - | - | 0.51±0.04bc | - |
| 2-Methylbutyraldehyde | 873 | 14.12±2.42a | 8.73±1.55c | 8.23±2.5cd | 4.27±0.89ef | 3.23±0.84ef | 14.99±1.91a | 10.57±2.42bc | 10.47±1.96bc | 12.43±3.17ab | 10.09±2.9bc | 5.57±0.83de | 13.35±2.5a | 5.20±0.79e | 5.51±0.65de | 2.06±0.28f |
| 3-Methyl-1-butanol | 925 | - | - | - | - | - | 0.70±0.05a | 0.63±0.05b | - | - | - | - | - | - | 0.58±0.00b | - |
| Pentanal | 957 | - | 4.87±0.91ab | 5.81±0.83ab | 5.94±2.7a | 5.00±1.14ab | - | - | - | - | - | 4.08±1.14b | - | 5.63±0.22ab | 4.35±0.27ab | 4.94±0.71ab |
| Isopropylacetone | 1010 | - | - | - | - | - | - | - | 0.29±0.1a | 0.19±0.22ab | - | 0.27±0.27a | - | 0.32±0.03a | 0.32±0.04a | 0.06±0.02b |
| 1-Penten-3-one | 1019 | 0.19±0.03cde | 0.07±0.01e | 0.42±0.10abc | 0.16±0.02de | 0.56±0.31a | 0.31±0.23bcd | 0.49±0.03ab | 0.43±0.25ab | 0.64±0.21a | 0.12±0.03de | 0.53±0.25ab | 0.57±0.06a | 0.52±0.16ab | 0.51±0.03ab | 0.48±0.02ab |
| Ethyl butyrate | 1033 | 0.02±0.00b | 0.02±0.00b | - | 0.08±0.04b | - | 0.22±0.06a | 0.19±0.02a | - | - | - | 0.16±0.19a | 0.21±0.02a | - | - | - |
| 2,3-Pentanedione | 1065 | 0.18±0.04ab | - | - | 0.17±0.09ab | 0.28±0.11a | 0.14±0.14b | 0.21±0.02ab | - | - | - | 0.25±0.14ab | - | - | - | - |
| Butyl acetate | 1072 | 0.07±0.02c | 0.10±0.07bc | - | - | 0.10±0.04bc | 0.13±0.10abc | 0.15±0.02abc | 0.15±0.06abc | 0.13±0.08abc | 0.20±0.23a | 0.15±0.11abc | 0.18±0.01ab | 0.16±0.10abc | 0.12±0.01abc | 0.09±0.00bc |
| Hexanal | 1075 | 2.28±0.13de | 1.35±0.27h | 2.95±0.19bc | 2.63±0.30cd | 3.61±0.23a | 1.55±0.14gh | 3.11±0.34b | 3.05±0.20bc | 2.78±0.39bc | 2.60±0.55cd | 1.57±0.23gh | 1.91±0.16efg | 2.81±0.68bc | 2.08±0.26ef | 1.68±0.07fgh |
| 4-Methyl-4-penten-2-one | 1094 | 0.32±0.05c | 0.20±0.12d | 0.04±0.01e | 0.34±0.03abc | - | 0.39±0.04abc | 0.43±0.05ab | 0.37±0.02abc | 0.33±0.04bc | 0.44±0.17a | 0.31±0.12cd | 0.31±0.06cd | 0.39±0.05abc | 0.39±0.05abc | 0.20±0.08d |
| 4-Methyl-3-penten-2-one | 1134 | 2.33±0.26bcde | 1.67±0.24fg | 1.85±0.08g | 2.06±0.18cdef | 2.46±0.27bc | 3.26±0.32a | 2.36±0.28bcde | 2.53±0.18b | 2.04±0.29defg | 2.41±0.23bcde | 2.00±0.26efg | 2.22±0.30bcde | 2.52±0.66b | 2.42±0.13bcd | 3.05±0.14a |
| β-Myrcene | 1141 | - | 0.30±0.01bc | - | - | - | - | 0.39±0.02ab | - | 0.10±0.10de | 0.48±0.04a | 0.25±0.02cd | 0.42±0.11a | 0.41±0.08a | 0.47±0.04a | 0.06±0.00e |
| 4-Methyl-2-heptanone | 1205 | 0.21±0.10ab | 0.19±0.07ab | - | 0.14±0.01b | - | - | - | 0.27±0.06a | - | - | 0.14±0.11ab | 0.16±0.09b | - | - | 0.14±0.03b |
| Ethyl caproate | 1223 | 0.72±0.30a | - | - | - | - | - | - | 0.35±0.06b | 0.28±0.08bc | 0.17±0.04bc | 0.19±0.04c | 0.19±0.03bc | - | - | - |
| 1-Pentanol | 1265 | 1.12±0.29bcde | 0.90±0.22def | 0.96±0.23def | 0.86±0.16ef | 0.85±0.24ef | 1.33±0.21bc | 0.77±0.10f | 0.81±0.19ef | 1.73±0.19a | 1.45±0.66ab | 1.24±0.09bcd | 1.06±0.10cdef | 1.13±0.24bcde | 0.89±0.30ef | 1.09±0.05bcdef |
| Octanal | 1285 | 0.23±0.05b | - | - | - | - | - | - | - | 0.34±0.12a | 0.19±0.06b | 0.17±0.03b | 0.20±0.13b | -b | - | 0.14±0.03 |
| 1-Octen-3-one | 1300 | - | - | - | - | - | - | 0.09±0.06c | - | 0.13±0.03bc | - | - | - | 0.15±0.05b | - | 0.31±0.04a |
| 2-Ethoxyethyl acetate | 1314 | 0.73±0.18ab | 0.47±0.13e | 0.43±0.01ef | 0.30±0.02fg | 0.24±0.07g | 0.28±0.04g | 0.27±0.05g | 0.44±0.02e | 0.61±0.07cd | 0.64±0.11bcd | 0.80±0.03a | 0.65±0.06bc | 0.81±0.10a | 0.53±0.06de | - |
| 2,3-Octanedione | 1330 | 0.42±0.14a | 0.20±0.07def | 0.34±0.04abc | 0.24±0.04cdef | 0.23±0.06cdef | 0.38±0.10ab | 0.20±0.02def | 0.17±0.02f | 0.37±0.09ab | 0.29±0.08bcde | 0.29±0.05bcd | 0.33±0.09abc | 0.32±0.04bc | 0.19±0.02ef | 0.31±0.04bc |
| 6-Methyl-5-hepten-2-one | 1347 | - | - | - | - | - | - | - | - | - | 0.61±0.05a | - | 0.16±0.06c | - | - | 0.37±0.18b |
| 1-Hexanol | 1373 | 0.18±0.03bc | 0.13±0.06ef | 0.11±0.01f | 0.14±0.01cdef | 0.14±0.04cdef | 0.13±0.02def | 0.15±0.01cdef | 0.17±0.02bcde | 0.18±0.05bcd | 0.14±0.02cdef | 0.15±0.03cdef | 0.15±0.02cdef | 0.17±0.05bcd | 0.20±0.03b | 0.43±0.01a |
| Nonanal | 1387 | 0.34±0.07bc | 0.13±0.03g | 0.28±0.07bcde | 0.13±0.03g | 0.15±0.04fg | 0.24±0.12cdef | 0.21±0.06defg | 0.25±0.11cdef | 0.37±0.05b | 0.52±0.10a | 0.17±0.07efg | 0.22±0.04defg | 0.33±0.05bc | 0.16±0.04fg | 0.29±0.15bcd |
| (Z)-3-Hexen-1-ol | 1400 | 0.19±0.06f | 0.21±0.02ef | 0.35±0.18bc | 0.18±0.01f | 0.18±0.02f | 0.19±0.02f | 0.24±0.05ef | 0.18±0.03f | 0.27±0.03cde | 0.38±0.06b | 0.37±0.05b | 0.25±0.05def | 0.32±0.03bcd | 0.17±0.03f | 0.96±0.02a |
| (Z)-2-Hexen-1-ol | 1426 | - | - | 0.12±0.05a | - | - | 0.07±0.01ab | - | - | - | - | - | - | - | 0.06±0.01b | - |
| (E)-2-Octenal | 1429 | 0.07±0.02b | - | - | 0.07±0.01c | 0.10±0.06bc | 0.08±0.01bc | 0.08±0.01bc | - | 0.10±0.02bc | - | - | - | 0.10±0.00bc | - | 0.24±0.03a |
| (E)-Linalool oxide (furanoid) | 1452 | 1.30±0.21de | 1.13±0.11ef | 1.79±0.18bc | 0.91±0.08fg | 0.77±0.21gh | 0.90±0.06fg | 1.30±0.19de | 1.56±0.16cd | 1.66±0.03h | 1.43±0.03h | 2.01±0.06b | 1.34±0.11de | 1.95±0.3b | 0.84±0.62fg | 4.04±0.53a |
| 1-Octen-3-ol | 1466 | 0.13±0.03bc | 0.07±0.01e | 0.10±0.01bcde | 0.10±0.01bcde | 0.08±0.02de | 0.09±0.02bcde | 0.13±0.02bcd | 0.09±0.01bcde | 0.12±0.02bcd | 0.10±0.00bcde | 0.06±0.01e | 0.09±0.02cde | 0.14±0.03b | 0.07±0.02e | 0.64±0.14a |
| 1-Heptanol | 1472 | 0.05±0.02bcd | 0.05±0.00bcd | 0.05±0.01bcde | 0.03±0.00efg | 0.03±0.01fg | 0.04±0.01cdef | 0.05±0.01bcd | 0.04±0.01defg | 0.06±0.01bc | 0.05±0.01bc | 0.06±0.01b | 0.06±0.01b | 0.06±0.02b | 0.03±0.00g | 0.17±0.00a |
| (E, E)-2,4-Heptadienal | 1475 | - | - | - | 0.18±0.05b | 0.14±0.03b | 0.21±0.03b | 0.15±0.02b | 0.11±0.01b | - | - | - | - | 0.19±0.02b | 0.08±0.00b | 1.54±0.49a |
| 2-Ethyl-1-hexanol | 1503 | - | 1.79±0.05de | 1.92±0.30de | 1.90±0.24de | 2.31±0.27d | 5.99±0.64a | 6.45±0.84a | 3.39±0.34bc | 3.61±0.42b | 2.93±0.08c | 2.24±0.16d | 1.88±0.08de | 3.27±0.39bc | 1.51±0.09ef | 1.03±0.06f |
| Benzaldehyde | 1527 | 1.01±0.12a | - | 1.01±0.07a | 0.43±0.11de | 0.49±0.16de | 0.61±0.33bcd | 0.72±0.07bc | 0.50±0.10de | 0.78±0.12b | 0.64±0.22bcd | - | 0.63±0.13bcd | 0.56±0.19cd | 0.30±0.07e | - |
| Linalool | 1563 | 2.34±0.19d | 2.04±0.05ef | 2.67±0.27c | 1.71±0.21gh | 1.52±0.21h | 1.91±0.21fg | 2.00±0.23ef | 2.20±0.21de | 3.13±0.16b | 2.38±0.07d | 1.94±0.12fg | 1.97±0.04ef | 2.67±0.20c | 1.61±0.14h | 6.53±0.07a |
| 1-Octanol | 1573 | 0.83±0.1b | 0.58±0.05cde | 0.56±0.16cdef | 0.46±0.06fg | 0.41±0.05g | 0.57±0.06cdef | 0.49±0.04efg | 0.58±0.11cdef | 0.66±0.10c | 0.68±0.11c | 0.64±0.07cd | 0.57±0.05cdef | 0.53±0.09def | 0.37±0.06g | 1.15±0.08a |
| Isophorone | 1592 | 0.59±0.06a | - | - | - | - | 0.16±0.02c | 0.21±0.02b | 0.13±0.04cd | 0.12±0.01d | 0.10±0.02de | 0.07±0.02e | - | 0.11±0.03de | - | - |
| Tea pyrrole | 1613 | 0.64±0.14def | 0.22±0.04h | 1.15±0.07a | 0.63±0.07def | 0.52±0.02fg | 0.45±0.09g | 0.21±0.05h | 0.52±0.06fg | 0.72±0.14bcd | 0.45±0.04g | 0.70±0.06cde | 0.79±0.07bc | 0.59±0.17ef | 0.83±0.04b | - |
| Hotrienol | 1626 | 0.31±0.16i | 0.87±0.09ef | 0.67±0.12gh | 0.62±0.03gh | 1.00±0.12de | 0.38±0.12i | 1.05±0.06d | 0.79±0.14fg | 0.62±0.14gh | 1.54±0.08c | 0.36±0.05i | 0.61±0.13h | 3.77±0.25a | 1.60±0.06c | 2.15±0.07b |
| Safranal | 1638 | 0.31±0.10b | 0.26±0.06bcd | 0.33±0.06b | 0.18±0.01e | 0.23±0.01cde | 0.22±0.07cde | 0.16±0.05e | 0.28±0.05bcd | 0.26±0.05bcd | 0.22±0.01cde | 0.30±0.08bc | 0.17±0.01e | 0.23±0.06cde | 0.20±0.01de | 0.58±0.08a |
| Benzeneacetaldehyde | 1657 | - | - | 0.80±0.37b | - | - | - | 0.70±0.49b | - | 1.60±0.84a | - | - | - | - | - | 0.60±0.07b |
| 1-Nonanol | 1674 | - | - | - | - | - | 0.26±0.18a | - | - | - | - | - | - | - | - | 0.13±0.01a |
| 1-Nonene | 1676 | 0.21±0.01a | - | - | 0.11±0.03c | - | - | - | - | - | - | - | - | 0.14±0.02b | - | - |
| α-Terpineol | 1708 | - | 0.09±0.01ef | 0.17±0.03bcd | - | 0.09±0.01f | 0.09±0.05ef | 0.13±0.07def | 0.12±0.04def | 0.14±0.03cdef | 0.22±0.04ab | 0.15±0.03cde | 0.10±0.02ef | 0.25±0.08a | 0.11±0.01def | 0.20±0.01abc |
| 2-Methyl-3-methyl-oxobutyrate | 1717 | 0.79±0.14a | - | - | 0.38±0.05bc | 0.26±0.04d | 0.48±0.08b | - | - | - | 0.39±0.06bc | 0.38±0.10bc | 0.40±0.09bc | 0.38±0.07bc | 0.36±0.06cd | - |
| Linalool oxide (pyranoid) | 1751 | - | 0.55±0.42bc | 0.14±0.09c | - | - | - | 1.10±0.27a | - | 0.19±0.13c | 0.50±0.27bc | 0.79±0.49ab | 0.34±0.05c | 0.8±0.65ab | 0.08±0.02c | 0.86±0.19ab |
| Methyl salicylate | 1779 | - | 0.46±0.14ef | - | 0.6±0.06cde | 0.73±0.04bc | - | - | 1.27±0.11a | 0.79±0.19b | 0.64±0.21bcd | 0.64±0.05bcd | 0.43±0.06f | 0.50±0.22def | 0.68±0.04bc | 1.11±0.28a |
| Methyl N-hydroxybenzimidate | 1787 | 1.01±0.53b | 0.75±0.23b | 0.96±0.59b | 0.82±0.15b | 1.63±1.57b | 0.83±0.19b | 0.79±0.09b | 0.87±0.27b | 0.84±0.13b | 0.99±0.49b | 1.17±0.11b | 0.84±0.30b | 1.02±0.44b | 0.81±0.25b | 0.59±0.21a |
| 1-(2-Butoxyethoxy)-ethanol | 1809 | - | - | - | - | - | 1.07±0.47a | 1.64±1.18a | 0.72±0.32a | - | - | - | - | - | - | - |
| Geraniol | 1818 | 4.73±0.72ef | 7.62±0.39d | 3.11±0.54hi | 2.28±0.34ij | 2.52±0.31ij | 4.30±0.57fg | 9.22±1.00c | 3.63±0.52gh | 4.06±0.41fg | 12.20±1.01a | 5.54±0.63e | 8.70±0.50c | 10.43±0.52b | 12.02±0.81a | 1.89±0.03j |
| 2,4-Dimethyl-benzaldehyde | 1870 | 8.64±0.71ab | 8.35±0.12ab | 6.54±0.43de | 8.00±0.95bc | 7.31±0.78cd | 7.88±0.77bc | 8.99±0.69a | 9.09±1.27a | 8.65±0.34ab | 6.77±0.27de | 6.23±0.66ef | 6.89±0.35de | 7.87±0.28bc | 5.46±0.97f | 9.27±0.23a |
| 4,5-Dimethyl-2-hepten-3-ol | 1881 | - | 1.81±0.14a | - | - | - | - | - | - | 1.57±0.26a | - | 1.71±0.65a | - | - | - | - |
| Propanoic acid, 2-methyl-, 1-(1,1-dimethylethyl)-2-methyl-1,3-propanediyl ester | 1881 | 2.46±0.03b | - | - | - | - | - | - | - | - | 3.40±0.09a | - | - | 2.48±0.70b | - | 0.21±0.08c |
| Benzyl alcohol | 1890 | 0.11±0.04e | 0.56±0.11ab | 0.13±0.00de | 0.19±0.09de | 0.26±0.07de | 0.15±0.01e | 0.31±0.12cd | 0.43±0.16bc | 0.43±0.08bc | 0.55±0.20ab | 0.59±0.16a | 0.60±0.15a | 0.61±0.04a | 0.33±0.07cd | 0.46±0.03abc |
| Phenylethyl alcohol | 1924 | 0.14±0.05gh | 0.27±0.04def | 0.43±0.09a | 0.08±0.01hi | 0.15±0.02g | 0.12±0.03ghi | 0.23±0.03f | 0.17±0.03g | 0.38±0.02ab | 0.36±0.04bc | 0.14±0.03g | 0.29±0.06de | 0.25±0.04ef | 0.08±0.02i | 0.31±0.04cd |
| β-Ionone | 1941 | 0.13±0.05bcdefg | 0.14±0.04bcdef | 0.18±0.07bc | 0.07±0.01g | 0.12±0.03cdefg | 0.11±0.03defg | 0.11±0.03efg | 0.11±0.04defg | 0.17±0.01bcd | 0.17±0.03bcde | 0.16±0.03bcde | 0.15±0.03bcde | 0.19±0.02b | 0.09±0.03fg | 1.63±0.13a |
| Jasmone | 1947 | - | 0.60±0.07bc | 0.60±0.14bc | 0.43±0.00de | 0.87±0.01a | - | 0.33±0.11fg | 0.37±0.11ef | 0.50±0.03cd | 0.47±0.10d | 0.30±0.01fg | 0.31±0.07fg | 0.70±0.07b | 0.25±0.09g | 0.91±0.12a |
| 1-Dodecanol | 1982 | 1.02±0.41c | 0.62±0.24cd | 0.59±0.26cd | 0.42±0.25cd | 2.06±0.95b | 2.48±0.65ab | 2.90±0.86a | 2.66±0.90ab | 0.66±0.70cd | 0.64±0.11cd | 0.57±0.42cd | 0.43±0.40cd | 0.50±0.12cd | 0.25±0.08d | 0.47±0.06cd |
| Nerolidol | 2064 | 0.17±0.06cd | 0.12±0.01cde | 0.44±0.13a | 0.14±0.03cde | 0.42±0.03a | 0.18±0.05c | 0.08±0.01e | 0.16±0.03cd | 0.26±0.04b | 0.33±0.10b | 0.10±0.04de | 0.18±0.03c | 0.26±0.02b | 0.11±0.02de | 0.31±0.00b |
| Cedrol | 2116 | 0.58±0.16a | - | - | - | - | - | 0.40±0.05b | 0.38±0.06b | - | - | - | - | - | - | - |
| n-Tridecanol | 2187 | - | - | - | - | - | 0.06±0.04c | 0.14±0.09ab | 0.08±0.03bc | - | - | 0.20±0.02a | - | - | - | - |
| Methyl hexadecanoate | 2225 | 0.31±0.17abc | 0.23±0.07cde | 0.21±0.05cde | 0.37±0.20ab | 0.24±0.19bcde | 0.16±0.03de | 0.14±0.04e | 0.16±0.03de | 0.17±0.05de | 0.33±0.11abc | 0.29±0.10abcd | 0.21±0.06cde | 0.24±0.10bcde | 0.13±0.02e | 0.40±0.03a |
| α-Cadinol | 2241 | - | 0.14±0.02b | - | - | - | - | - | - | - | 0.18±0.03a | - | - | - | 0.07±0.01c | - |
| Diethyl phthalate | 2387 | 0.67±0.48a | 0.70±0.25a | 0.78±0.40a | - | 0.55±0.58ab | 0.12±0.08b | - | - | - | - | - | - | - | 0.91±0.36a | 0.79±0.09a |
| 2,3-Dihydrobenzofuran | 2410 | 0.45±0.13cdef | 0.52±0.04cde | - | 0.38±0.04def | 0.54±0.17bcd | 0.30±0.11fg | 0.33±0.11f | 0.59±0.09abc | 0.34±0.02ef | 0.38±0.19def | 0.71±0.16ab | 0.58±0.24abc | 0.53±0.20cd | 0.75±0.09a | 0.11±0.00g |
| Diisobutyl phthalate | 2561 | - | 2.91±0.55b | 3.68±0.46a | 2.06±0.09cde | 2.36±0.60bc | 1.93±0.25cde | 1.43±0.29e | 2.52±0.44bc | 1.91±0.15cde | 2.16±0.23c | 2.11±0.68cd | 1.51±0.24de | 2.06±0.46cde | 2.04±0.75cde | - |

Note: ZC108, 'Zhongcha 108'; ZN805, 'Zhenong 805'; ZN801, 'Zhenong 801'; ZN901, 'Zhenong 901'; ZN301, 'Zhenong 301'; ZN302, 'Zhenong 302'; JKZ, 'Jiukengzao'; XX1, 'Xiaoxiang 1'; FDDB, 'Fuding Dabaicha'; ZHDB, 'Zhenghe Dabaicha'; MZ, 'Mei Zhan'; FJSX, 'Fujian Shuixian'; PYTZ, 'Pingyang Tezao'. Retention index (RI) was calculated from the retention times of the volatiles and a homologous series of n-alkanes (C7-C30). Different letters in a row indicated significant difference at *p*<0.05. -. not detected.

**Table S15**. Relative odor activity values of co-owned volatiles screened out from PYHT tea prepared from the shoots of different cultivars.

| Volatiles | Threshold  (μg/L) | Descriptions | Relative odor activity values | | | | | | | | | | | | | | |
| --- | --- | --- | --- | --- | --- | --- | --- | --- | --- | --- | --- | --- | --- | --- | --- | --- | --- |
|  |  |  | ZC108 | ZN805 | ZN801 | ZN901 | ZN301 | ZN302 | JKZ | 5-21 | 1-35 | XX1 | FDDB | ZHDB | MZ | FJSX | PYTZ |
| 2-Methylbutyraldehyde | 1 | Malty | 282.33 | 174.52 | 164.52 | 85.42 | 64.60 | 299.89 | 211.42 | 209.30 | 248.64 | 201.73 | 111.42 | 267.01 | 104.03 | 110.19 | 41.27 |
| Hexanal | 4.5 | Grassy, green, fresh, fatty | 10.15 | 6.01 | 13.12 | 11.67 | 16.04 | 6.87 | 13.82 | 13.56 | 12.38 | 11.56 | 6.99 | 8.47 | 12.47 | 9.26 | 7.48 |
| Nonanal | 1.1 | Floral, fatty, green, lemon-like | 6.19 | 2.33 | 5.09 | 2.30 | 2.75 | 4.41 | 3.84 | 4.56 | 6.78 | 9.51 | 3.03 | 3.98 | 5.92 | 2.91 | 5.34 |
| Safranal | 3 | Woody, spicy, phenolic | 2.09 | 1.76 | 2.17 | 1.20 | 1.53 | 1.47 | 1.05 | 1.87 | 1.74 | 1.47 | 1.97 | 1.13 | 1.54 | 1.35 | 3.85 |
| 2,4-Dimethyl-benzaldehyde | 0.2 | Bitter almond flavor | 863.76 | 835.24 | 653.58 | 800.11 | 731.13 | 788.16 | 898.81 | 908.53 | 865.12 | 676.95 | 622.66 | 689.01 | 786.62 | 546.25 | 926.58 |
| 1-Pentanol | 3.9 | Balsamic | 5.74 | 4.63 | 4.90 | 4.42 | 4.35 | 6.81 | 3.92 | 4.15 | 8.87 | 7.41 | 6.36 | 5.46 | 5.78 | 4.57 | 5.61 |
| 1-Hexanol | 5.6 | Green, grassy | 0.64 | 0.45 | 0.39 | 0.52 | 0.50 | 0.48 | 0.53 | 0.59 | 0.63 | 0.49 | 0.53 | 0.53 | 0.61 | 0.70 | 1.55 |
| (Z)-3-Hexen-1-ol | 70 | Green, leafy, grassy | 0.05 | 0.06 | 0.10 | 0.05 | 0.05 | 0.06 | 0.07 | 0.05 | 0.08 | 0.11 | 0.11 | 0.07 | 0.09 | 0.05 | 0.28 |
| (E)-Linalool oxide (furanoid) | 60 | Flowery | 0.43 | 0.38 | 0.60 | 0.30 | 0.26 | 0.30 | 0.43 | 0.52 | 0.15 | 0.14 | 0.67 | 0.45 | 0.65 | 0.28 | 1.35 |
| 1-Octen-3-ol | 1 | Mushroom | 2.66 | 1.39 | 2.01 | 1.96 | 1.59 | 1.88 | 2.52 | 1.89 | 2.38 | 2.04 | 1.27 | 1.79 | 2.81 | 1.36 | 12.81 |
| 1-Heptanol | 400 | Fruity, soapy | 0.00 | 0.00 | 0.00 | 0.00 | 0.00 | 0.00 | 0.00 | 0.00 | 0.00 | 0.00 | 0.00 | 0.00 | 0.00 | 0.00 | 0.01 |
| Linalool | 0.22 | Floral, sweet, grape-like, woody | 213.05 | 185.27 | 242.35 | 155.09 | 138.52 | 173.74 | 181.38 | 200.05 | 285.00 | 216.01 | 176.62 | 178.78 | 242.80 | 146.30 | 593.55 |
| 1-Octanol | 0.022 | Green, citrus, fatty, coconut-like | 753.04 | 524.35 | 508.77 | 415.41 | 370.22 | 516.09 | 442.70 | 523.47 | 598.14 | 618.15 | 586.28 | 516.50 | 485.76 | 335.14 | 1048.54 |
| Hotrienol | 110 | Floral, sweet, grassy | 0.06 | 0.16 | 0.12 | 0.11 | 0.18 | 0.07 | 0.19 | 0.14 | 0.11 | 0.28 | 0.07 | 0.11 | 0.69 | 0.29 | 0.39 |
| Geraniol | 7.5 | Rose-like, sweet, honey-like | 12.61 | 20.32 | 8.31 | 6.08 | 6.73 | 11.46 | 24.58 | 9.67 | 10.83 | 32.53 | 14.77 | 23.19 | 27.82 | 32.06 | 5.04 |
| Benzyl alcohol | 100 | Bitter almond | 0.02 | 0.11 | 0.03 | 0.04 | 0.05 | 0.03 | 0.06 | 0.09 | 0.09 | 0.11 | 0.12 | 0.12 | 0.12 | 0.07 | 0.09 |
| Phenylethyl alcohol | 390 | Floral, rose-like | 0.01 | 0.01 | 0.02 | 0.00 | 0.01 | 0.01 | 0.01 | 0.01 | 0.02 | 0.02 | 0.01 | 0.02 | 0.01 | 0.00 | 0.02 |
| 1-Dodecanol | 1000 | Earthy, soapy, waxy, fatty, honey, coconut | 0.02 | 0.01 | 0.01 | 0.01 | 0.04 | 0.05 | 0.06 | 0.05 | 0.01 | 0.01 | 0.01 | 0.01 | 0.01 | 0.00 | 0.01 |
| Nerolidol | 10 | Flowery | 0.34 | 0.25 | 0.88 | 0.28 | 0.83 | 0.36 | 0.17 | 0.32 | 0.53 | 0.66 | 0.20 | 0.36 | 0.52 | 0.21 | 0.61 |
| Ethyl acetate | 5 | fruity | 3.58 | 1.95 | 6.27 | 5.31 | 7.22 | 7.00 | 6.06 | 5.43 | 6.72 | 1.74 | 5.85 | 5.87 | 8.42 | 5.47 | 2.83 |
| Methyl hexadecanoate | 1000 | Oily, waxy, fatty | 0.01 | 0.00 | 0.00 | 0.01 | 0.00 | 0.00 | 0.00 | 0.00 | 0.00 | 0.01 | 0.01 | 0.00 | 0.00 | 0.00 | 0.01 |
| 1-Penten-3-one | 1 | Pungent | 3.80 | 1.46 | 8.37 | 3.22 | 11.17 | 6.27 | 9.72 | 8.53 | 12.76 | 2.32 | 10.54 | 11.49 | 10.38 | 10.10 | 9.57 |
| 4-Methyl-3-penten-2-one | 200 | Honey-like, card board-like, nutty, woody | 0.23 | 0.17 | 0.16 | 0.21 | 0.25 | 0.33 | 0.24 | 0.25 | 0.20 | 0.24 | 0.20 | 0.22 | 0.25 | 0.24 | 0.30 |
| 2,3-Octanedione | 3 | Milk, buttery, cooked tasting | 2.80 | 1.32 | 2.24 | 1.60 | 1.53 | 2.54 | 1.33 | 1.13 | 2.44 | 1.91 | 1.93 | 2.19 | 2.11 | 1.25 | 2.06 |
| *β*-Ionone | 0.01 | Violet-like, raspberry, floral | 256.92 | 283.87 | 361.24 | 142.15 | 238.65 | 225.79 | 215.11 | 226.01 | 340.55 | 332.99 | 315.46 | 319.54 | 370.78 | 184.71 | 3250.39 |
| Methyl N-hydroxybenzimidate | n.f. | n.f. | - | - | - | - | - | - | - | - | - | - | - | - | - | - | - |

Note: The remaining 20 volatiles were not listed, as the odor threshold was not consulted.
